# Regulation of the apico-basolateral trafficking polarity of the homologous copper-ATPases ATP7A and ATP7B

**DOI:** 10.1242/jcs.261258

**Published:** 2023-11-30

**Authors:** Ruturaj, Monalisa Mishra, Soumyendu Saha, Saptarshi Maji, Enrique Rodriguez-Boulan, Ryan Schreiner, Arnab Gupta

**Affiliations:** ^1^ Department of Biological Sciences, Indian Institute of Science Education and Research Kolkata, Mohanpur, West Bengal 741246, India; ^2^ Department of Ophthalmology, Margaret Dyson Vision Research Institute, Weill Cornell Medicine, New York, NY 10021, USA; ^3^ Division of Regenerative Medicine, Department of Medicine, Weill Cornell Medicine, New York, NY 10021, USA

**Keywords:** ATP7A, ATP7B, Copper-ATPases, Apico-basolateral polarity, Adaptor protein-1, MDCK

## Abstract

The homologous P-type copper-ATPases (Cu-ATPases) ATP7A and ATP7B are the key regulators of copper homeostasis in mammalian cells. In polarized epithelia, upon copper treatment, ATP7A and ATP7B traffic from the trans-Golgi network (TGN) to basolateral and apical membranes, respectively. We characterized the sorting pathways of Cu-ATPases between TGN and the plasma membrane and identified the machinery involved. ATP7A and ATP7B reside on distinct domains of TGN in limiting copper conditions, and in high copper, ATP7A traffics to basolateral membrane, whereas ATP7B traverses common recycling, apical sorting and apical recycling endosomes *en route* to apical membrane. Mass spectrometry identified regulatory partners of ATP7A and ATP7B that include the adaptor protein-1 complex. Upon knocking out pan-AP-1, sorting of both Cu-ATPases is disrupted. ATP7A loses its trafficking polarity and localizes on both apical and basolateral surfaces in high copper. By contrast, ATP7B loses TGN retention but retained its trafficking polarity to the apical domain, which became copper independent. Using isoform-specific knockouts, we found that the AP-1A complex provides directionality and TGN retention for both Cu-ATPases, whereas the AP-1B complex governs copper-independent trafficking of ATP7B solely. Trafficking phenotypes of Wilson disease-causing ATP7B mutants that disrupts putative ATP7B–AP1 interaction further substantiates the role of AP-1 in apical sorting of ATP7B.

## Teaser

**Summary:** The adaptor protein-1 (AP-1) isoforms AP-1A and AP-1B govern the apico-basolateral trafficking polarities of the homologous copper-ATPases ATP7A and ATP7B at the trans-Golgi network.

## INTRODUCTION

Copper is a crucial micronutrient for all eukaryotic organisms (Mustafa and Alsharif, 2018; Olivares and Uauy, 1996). Copper is a redox-active metal (Koppenol and Bounds, 2011; Tsang et al., 2021). Its intracellular levels need to be tightly regulated as copper accumulation causes severe toxicity (Harris, 1992). Cellular copper is primarily regulated by Golgi-localizing copper-transporting ATPases (Cu-ATPases), ATP7A and ATP7B, which are highly homologous P-type ATPases, that pump copper into the lumen of the trans-Golgi network (TGN) where various proteins utilize it as a co-factor for their maturation (Hung et al., 1997; Sato and Gitlin, 1991; Setty et al., 2008). Upon elevated copper levels, these Cu-ATPases are redistributed by vesicular trafficking from TGN to the plasma membrane, where they export excess copper out of the cell (La Fontaine and Mercer, 2007; Lutsenko et al., 2007; Voskoboinik and Camakaris, 2002). Pathogenic variants in ATP7A, which is ubiquitously expressed and abundant in enterocytes, lead to systemic deficiency of copper, causing Menkes disease (Tümer and Møller, 2010). In contrast, pathogenic variants in ATP7B lead to copper accumulation in liver, kidney and brain, leading to Wilson disease, which is characterized by liver cirrhosis and neurological symptoms (Cox, 1999; Terada et al., 1998).

Lower organisms that precede chordate evolution have only one copper ATPase. Evolutionary divergence from a single copper ATPase to ATP7A and ATP7B is first observed in fish (Gupta and Lutsenko, 2012). Human ATP7A and ATP7B share ∼65% sequence identity, with highly conserved domains that include the cytoplasmic N-terminus, which harbors six copper-binding domains containing the sequence MxCxxC, the nucleotide-binding domain, the phosphorylation domain, the actuator domains and the C-terminus (Gupta et al., 2018). Under normal physiological conditions, as cellular copper increases, the six metal-binding domains in the N-terminal sequester copper. This causes structural changes in the proteins that promote their redistribution to the plasma membrane to export excess intracellular copper (LeShane et al., 2010; Lim et al., 2006). In polarized epithelial cells exposed to elevated copper, both transporters leave the TGN, with ATP7A directed to the basolateral surface and ATP7B to the apical surface (Greenough et al., 2004; Nyasae et al., 2014).

Studies carried out on the epithelial MDCK cell line and other epithelial cell lines have identified the TGN as the compartment where apical and basolateral plasma membrane proteins are segregated into distinct routes to the plasma membrane (Apodaca et al., 2012; Klemm et al., 2009; Mostov and Cardone, 1995; Rodriguez-Boulan et al., 2005; Rodriguez-Boulan and Macara, 2014; Yeaman et al., 2004; Zurzolo and Simons, 2016). During transport to the cell surface, some plasma membrane proteins might traffic through endosomal compartments, such as common recycling endosomes (CREs), apical recycling endosomes (AREs) and apical sorting endosomes (ASEs), where additional sorting events might take place (Ang et al., 2004; Fölsch et al., 2009; Gonzalez and Rodriguez-Boulan, 2009; Thuenauer et al., 2014; Weisz and Rodriguez-Boulan, 2009). Transport along these routes is directed by apical signals such as N-glycans (Scheiffele et al., 1995), O-glycans (Yeaman et al., 1997) and GPI anchors (Lisanti et al., 1989; Powell et al., 1991), and basolateral signals that resemble tyrosine and di-leucine endocytic motifs in structure (Lipardi et al., 2002). A variety of molecules have been postulated to mediate these trafficking processes, such as clathrin (Deborde et al., 2008), the clathrin adaptor AP-1A and AP-1B complexes (Cancino et al., 2007; Fölsch et al., 1999; Gan et al., 2002; Gonzalez and Rodriguez-Boulan, 2009; Gravotta et al., 2007; 2012), microtubule motors (Jaulin et al., 2007; Perez Bay et al., 2013; Weisz and Rodriguez-Boulan, 2009) and regulators of the actin cytoskeleton (Braiterman et al., 2009; Gupta et al., 2016; Salvarezza et al., 2009).

Limited trafficking studies have been carried out on the polarized trafficking of ATP7A and ATP7B. ATP7A has been mostly studied in non-epithelial cell models like HeLa and HEK293 cells (Holloway et al., 2013; Yi and Kaler, 2015; Zhu et al., 2016). Hubbard and coworkers have utilized the liver epithelial cell line WIF-B as a model to study apical trafficking signals in ATP7B (Guo et al., 2005; Braiterman et al., 2011; Braiterman et al., 2014; Gupta et al., 2016). Braiterman et al. found that the Wilson disease-causing mutation ATP7B-N41S results in the mislocalization of ATP7B to the basolateral surface (Braiterman et al., 2009). The N41S mutation lies within a stretch of 9 amino acids that was determined to be essential for apical targeting of ATP7B in polarized hepatocytes; the deletion of which resulted in basolateral targeting of ATP7B (Braiterman et al., 2009). In neurons, the YxxФ motif at the C-terminus of ATP7B has been shown to be critical in maintaining somatodendritic polarity and TGN localization (Jain et al., 2015). The bona fide clathrin-interacting dileucine motif within the DKSWLLL stretch on the C-terminus of ATP7B has also been shown to be crucial for its copper-dependent trafficking (Lalioti et al., 2014). Similarly, a di-leucine motif at the C-terminus of ATP7A was found to be important for its internalization and retrograde transport from the plasma membrane to TGN (Zhu et al., 2016). Myosin Vb and its effector Rab-GTPase Rab11 (herein referring to Rab11a) were shown to be essential for the apical trafficking of ATP7B (Gupta et al., 2016). Fundamental questions remain unanswered on the differential regulation, directionality and the sorting compartments involved in the polarized trafficking of ATP7A and ATP7B.

We investigated the intracellular pathways, signals and mechanisms directing ATP7A and ATP7B to opposite poles of epithelial cells using the Madin–Darby canine kidney (MDCK) cell line. These two proteins are ideally suited for these studies as they are highly homologous and exhibit synchronized trafficking in response to the same ligand (i.e. copper). The absence or chelation of copper naturally acts as their Golgi-exit blocker as they are primarily Golgi-resident proteins. Our results identify intracellular compartments involved in the trafficking of these two proteins and demonstrate the role of clathrin adaptors AP-1A and AP-1B in their polarized sorting.

## RESULTS

### ATP7A and ATP7B traffic to basolateral and apical surfaces respectively in polarized MDCK cells

**Fig. 1.**
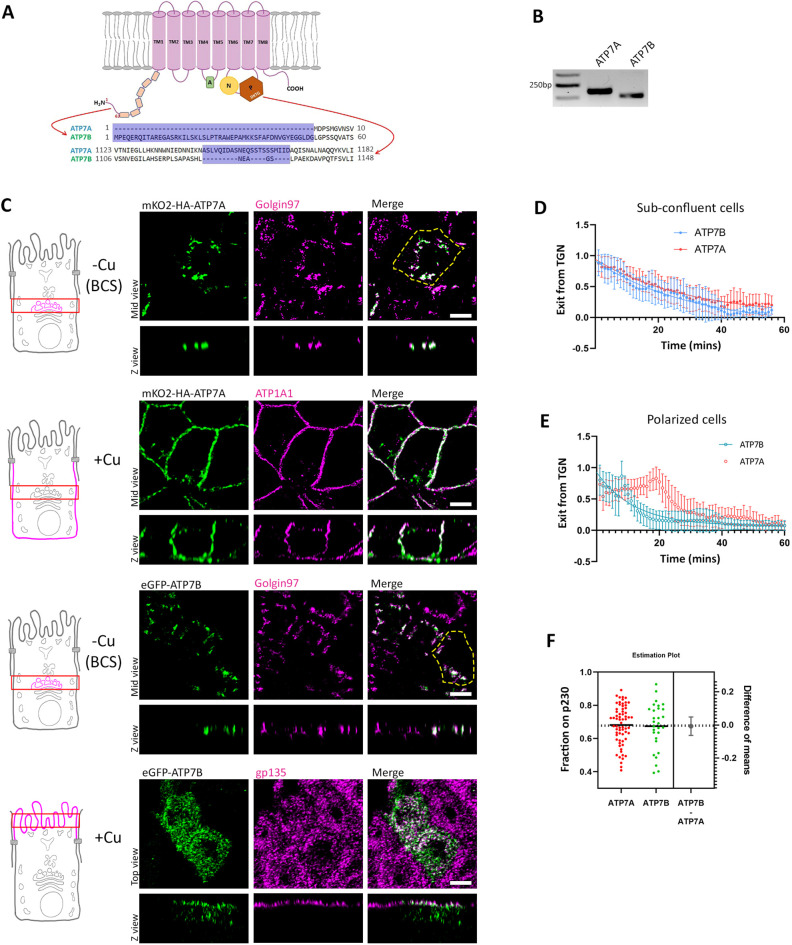
**ATP7A and ATP7B are homologous proteins and are expressed in MDCK cells but traffic to different surfaces in response to copper.** (A) Illustration showing the structure of the Cu-ATPases with major sequence differences between ATPA and ATP7B drawn out. (B) Transcript levels of dog ATP7A and ATP7B showing the expression of both the transporters in MDCK cells. Gel representative of three repeats. (C) Polarized MDCK cells showing localization of transfected mKO2–HA–ATP7A and eGFP–ATP7B in copper-depleted and copper-treated conditions. Under copper-depleted conditions, both proteins localize at the TGN marked by golgin-97. Upon copper treatment, ATP7A traffics to the basolateral surface (marked by ATP1A1) and ATP7B traffics to the apical surface (marked by gp135). The yellow dashed line indicates the cell boundary. (D) Fitted curve of decreased pixel count for both the Cu-ATPases, mKO2–HA–ATP7A and eGFP–ATP7B, in response to copper in sub-confluent MDCK cells revealing similar dispersion and Golgi-exit rates. Sample size (*N*) for ATP7A: 20, ATP7B: 13. (E) Fitted curve of decreased pixel count of both the Cu-ATPases, mKO2–HA–ATP7A and eGFP–ATP7B, in response to copper in polarized MDCK cells. Sample size (*N*) for ATP7A: 20, ATP7B: 25. (F) Colocalization quantification (Manders’ colocalization coefficient) for transfected mKO2–HA–ATP7A and eGFP–ATP7B with the TGN marker p230 (Fixed cells treated with 2.5 µM CuCl_2_ for 30 min) reveals their equal sensitivity to copper. Results in D–F are mean±s.d. Scale bars: 5 µm.

ATP7A and ATP7B are highly homologous proteins with conserved functional domains. However, they exhibit sequence dissimilarity in two specific regions – at the extreme N-terminus and at a flexible unstructured loop at the nucleotide-binding domain (Fig. 1A). Some cell types co-express both the copper-ATPases. Both individuals with Menkes disease and those with Wilson disease exhibit copper accumulation in tubules of the nephron (Moore and Cox, 2002). Barnes and co-workers have shown that HEK293, a cell line derived from human embryonic kidney expresses both ATP7A and ATP7B (Barnes et al., 2009). We found that MDCK cells express transcripts for both the copper transporters (Fig. 1B). We have previously shown that copper levels do not affect the endogenous expression levels of ATP7A and ATP7B, which corroborates findings from other groups (Das et al., 2022; Barnes et al., 2009; Leonhardt et al., 2009). Owing to their being no commercial antibodies against dog ATP7A and ATP7B, we expressed fluorescently tagged ATP7A and ATP7B (i.e. mKO2–HA–ATP7A and eGFP–ATP7B). mKO2 is a monomeric orange fluorescent protein with an excitation peak of 552 nm and an emission peak of 565 nm. We used salt of tetrathiomolybdate (TTM, 25 µM) or bathocuproinedisulfonic acid (BCS, 50 µM) as copper chelators to mimic low copper conditions; CuCl_2_ (10–50 µM) treatment in culture medium was used to elevate intracellular copper levels. Using inductively coupled plasma mass spectrometry (ICP-MS) the copper level in the basal medium was determined to be ∼0.7±0.2 µM (mean±s.d.).

To study the copper-induced trafficking routes and regulation of the two homologous Cu-ATPases, we expressed tagged mKO2–HA–ATP7A and eGFP–ATP7B in MDCK-II cells. Cells were allowed to completely polarize for 3–4 days on permeable filters. We found that at copper chelated and basal copper levels, both proteins localized at the TGN marked by golgin-97 (GOLGA1). Upon copper treatment (50 µM CuCl_2_ for 2 h) ATP7A trafficked to the basolateral surface marked by ATP1A1 (a Na/K-ATPase subunit), whereas ATP7B localized to the apical surface marked by gp135 (PODXL) (Fig. 1C). Given that both proteins share high sequence similarity (∼65%) with all the conserved signature domains of Cu-ATPases, we wanted to see whether they are comparably responsive to copper treatment. We analyzed their exit rate and sensitivity in terms of their trafficking from TGN. Golgi exit rate was calculated by loss of mKO2 or eGFP fluorescence from the TGN after copper treatment (see Materials and Methods). We treated the cells with copper (50 µM CuCl_2_) and imaged continuously at 60 s intervals (Movies 1 and 2). Upon plotting the signal distribution, we found both the transporters exhibited a similar Golgi-exit rate (Fig. 1D). We calculated the total fluorescence in a cell and found that it did not change over time or on copper treatment, hence excluding the possibility of fluorescence quenching. Also, we did not observe any significant drop in fluorescence over time in untreated (control) cells expressing the fluorescent-tagged ATP7A and ATP7B, thus excluding possible quenching-related intensity drop. They showed comparable *k* values (i.e. ATP7A, 0.03386; ATP7B, 0.03225) (Fig. S1A,B). Interestingly we observed very different kinetics in polarized cells for ATP7A. Upon copper treatment, the peak of the distribution of TGN exit for ATP7A occurred at ∼20 min and then followed a one-phase decay (Fig. 1E; Fig. S1C, Movie 3). This pattern corroborates with a previous report by Jaulin et al. (Jaulin et al., 2007). However, ATP7B followed a pattern similar to that in subconfluent cells, but showing a faster rate than them (Fig. 1E; Fig. S1D, Movie 4). We also checked for the sensitivity of these proteins towards copper. We treated the cells with low copper (2.5 µM CuCl_2_) to facilitate Golgi exit and measured the amount of ATP7A or ATP7B at the TGN, marked by p230 (GOLGA4). Calculating the Manders' colocalization coefficient (MCC), we found that both copper exporters are stimulated to leave the TGN by identical copper concentrations (Fig. 1F; Fig. S1E). We confirmed that co-expressing ATP7A and ATP7B in polarized MDCK cells did not affect their respective copper-responsive localization (50 µM CuCl_2_ for 2 h) (Fig. S1F).

### Copper level regulates segregation of ATP7A and ATP7B into distinct TGN subdomains

ATP7A and ATP7B localize at the TGN in basal and depleted copper conditions (Das et al., 2022; Petris et al., 1996). We asked whether ATP7A and ATP7B colocalize within the TGN domains and whether copper-dependent polarized trafficking of ATP7A and ATP7B is preceded by changes in their localization within the TGN. The copper ATPases harbor six copper-binding motifs (CxxC) on their cytosolic N-terminus, and copper saturation of these sites acts as a trigger for their TGN exit (Hasan et al., 2012; Strausak et al., 1999). We postulated that gradual saturation of the six CxxC motifs with copper might correlate with changes in the localization of ATP7A and ATP7B within the TGN that precede their TGN exit via separate routes. To study this point, we varied the copper levels in the cell. We used (1) basal, (2) extracellular copper chelation by means of BCS to arrest copper uptake (25 µM BCS, 4 h) and (3) intracellular copper depletion by means of TTM (10 µM TTM, 4 h), to mimic basal copper, mild and severe copper chelation, respectively, in the cell.

**Fig. 2.**
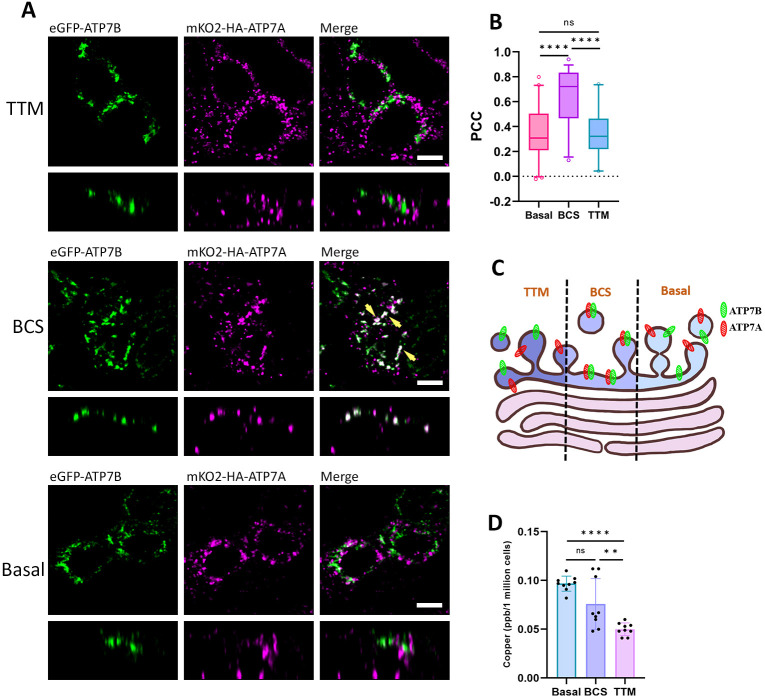
**ATP7A and ATP7B occupy distinct domains at TGN under various copper levels.** (A) Polarized MDCK cells showing localization of eGFP–ATP7B (green) and mKO2–HA–ATP7A (magenta) under limiting copper conditions created by treatment of TTM and BCS compared to basal level. Both the Cu-ATPases occupy distinct domains under TTM and basal conditions, unlike in BCS-treated cells, where they colocalize (indicated by yellow arrowheads). (B) Colocalization quantification (Pearson's correlation coefficient) of transfected mKO2–HA–ATP7A with eGFP–ATP7B under basal, and TTM- and BCS-treated conditions are demonstrated by box plot. The box represents the 25th to 75th percentiles. The whiskers show the data points within a range of 1.5× interquartile range (IQR) from the first and third quartile. Sample size (*N*) for basal: 41, BCS: 38, TTM: 20. (C) Schematic of our findings showing ATP7A (marked in red) and ATP7B (marked in green) occupying distinct and juxtaposed positions under the said conditions. (D) Measurement of intracellular copper level by ICP-MS in basal, and BCS- and TTM-treated conditions. This shows higher copper chelation in TTM-treated cells than in BCS- and basal cells. Results in are mean±s.d. (*n*=3). Scale bars: 5 µm.

We found that, under basal copper levels, ATP7A and ATP7B localize into distinct TGN subdomains without any signal overlap. Interestingly, in BCS-treated cells we found that ATP7A and ATP7B colocalize. However, surprisingly upon severe copper depletion through treatment with the intracellular copper chelator, we found both the proteins at distinct non-colocalizing positions on the TGN (Fig. 2A,B). This indicates that the slightest change in copper concentration can dictate the intra-TGN localization of ATP7A and ATP7B. Furthermore, our findings suggest that the two Cu-ATPases colocalize at a sub-basal copper condition and are uniquely located on distinct TGN domains in condition of severe copper chelation and basal copper levels. Upon simultaneous co-staining of ATP7A and ATP7B with TGN marker p230, we found a similar pattern. ATP7A and ATP7B showed a higher level of colocalization with p230 under the BCS condition compared to the TTM-treated and basal conditions (Fig. S2). The schematic (Fig. 2C) illustrates our findings. We confirmed the three conditions of basal copper, mild copper chelation (+BCS) and severe copper chelation (+TTM) by measuring the concentration of intracellular copper by ICP-MS (Fig. 2D).

### Post-TGN trafficking of ATP7B but not ATP7A involves intermediate endosomal compartments

**Fig. 3.**
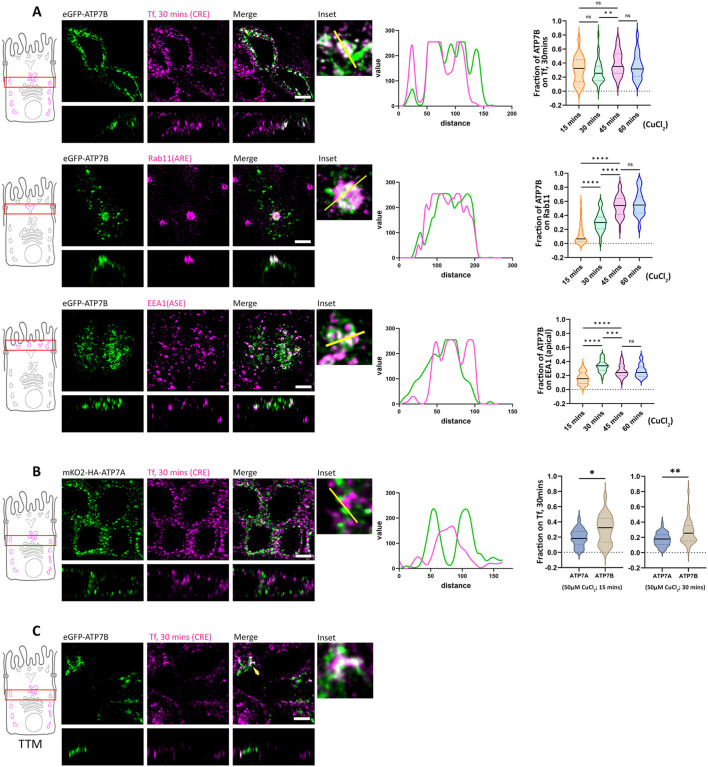
**ATP7B traffics via different compartments whereas ATP7A does not traverse any intermediate compartment.** (A) Polarized MDCK cells showing localization of transfected eGFP–ATP7B within various intermediate endosomes as it traffics in response to copper. Insets show zoomed images, and line profiles show pixel overlap on marked endosomes. Colocalization quantifications (Manders' colocalization coefficient) of transfected eGFP–ATP7B after a 30 min internalization Tf in all cases, with Rab11 and EEA1 at different time points of copper treatment (50 µM CuCl_2_) are shown on the right (*N*>30 for each condition). ATP7B traffics via CREs (30 min Tf–633 internalization), AREs (Rab11-positive compartments) and ASEs (EEA1-positive compartments). (B) Polarized MDCK cells showing localization of transfected mKO2–HA–ATP7A with CREs and BSEs (30 min Tf internalization) as it traffics in response to copper. ATP7A has very low overlap with CREs and BSEs (30 min Tf internalization). Colocalization quantification (Manders' colocalization coefficient) of transfected mKO2–HA–ATP7A versus eGFP–ATP7B with internalized Tf (30 min) at 15 min and 30 min copper treatment [Sample size (*N*) for (i) 15 min CuCl_2_ treatment; ATP7A: 60, ATP7B: 22, and (ii) 30 min CuCl_2_ treatment; ATP7A: 50, ATP7B: 34]. Cells were treated with 50 µM CuCl_2_ for 30 min for A and B. (C) Confocal image of polarized MDCK cells showing presence of eGFP–ATP7B (green) in CREs (magenta; 30 min Tf internalization) under TTM-treated conditions, indicating copper-independent trafficking of ATP7B (indicated by arrowhead). Violin plots show the median and quartiles, with a dotted line at 0. *P<0.05, **P<0.01, ***P<0.001, ****P<0.0001, ns; not significant (Mann–Whitney *U*-test/Wilcoxon rank-sum test). Scale bars: 5 µm.

Extensive studies on polarized protein trafficking in MDCK cells have identified endosomal compartments that are traversed by plasma membrane-targeted proteins after leaving the TGN. These compartments contribute to their apical-basolateral sorting of the trafficking proteins. In addition to their roles in endocytic recycling, common recycling endosomes (CREs), apical recycling endosomes (AREs), apical and basolateral sorting endosomes (ASEs and BSEs, respectively) have been implicated as post-TGN trafficking and sorting compartments (Ang et al., 2004; Fölsch et al., 2009; Gonzalez and Rodriguez-Boulan, 2009; Perez Bay et al., 2015; Perret et al., 2005; Thompson et al., 2007; Thuenauer et al., 2014; Weisz and Rodriguez-Boulan, 2009). We determined the copper-induced post-TGN trafficking itineraries of the Cu-ATPases by studying their colocalization with markers of CREs, AREs and ASEs. We treated polarized MDCK cells expressing eGFP–ATP7B with copper (50 µM CuCl_2_) and fixed them. We found that post TGN exit, ATP7B colocalizes with CREs, marked by transferrin (Tf) internalization for 30 min at four distinct time points of copper treatment (i.e. 15, 30, 45 and 60 min). We observed a comparable colocalization between ATP7B and 30 min internalized Tf at all the four time points of copper treatment (Fig. 3A, top right panel). We also found ATP7B in Rab11- and EEA1-positive compartments, suggesting the apical route of ATP7B involves both AREs, a slow recycling compartment (marked by Rab11), and ASEs, a fast-recycling compartment (marked by EEA1) (Fig. 3A). With increasing length of copper treatment there was an increase in ATP7B–Rab11 colocalization that reached a steady state at 45 min (Fig. 3A, middle panel). Similarly, ATP7B–EEA1 colocalization peaked at 30 min and then attained a steady state at 45 and 60 min of copper treatment (Fig. 3A, bottom panel). We also confirmed the presence of ATP7B in ASE by co-staining it with 5 min apically internalized wheat germ agglutinin (WGA), which labels ASEs (Fig. S3A), which further strengthens the evidence of fast recycling of ATP7B. In contrast with a report by Nyasae et al., we did not observe ATP7B in BSEs after 5 min of Tf internalization (Fig. S3B) (Nyasae et al., 2014) or after 3 min basolateral dextran uptake (Fig. S3C). For ATP7A, which is targeted basolaterally, we observed minimal colocalization with internalized Tf (30 min) upon copper treatment (Fig. 3B). We also failed to observe colocalization of ATP7A with AREs and ASEs (Rab11- and EEA1-positive compartments) or BSEs (marked by 5 min internalized Tf) (Fig. S3D–F). To summarize, our findings suggest that the apical route of ATP7B involves post-TGN passage through CREs, AREs and ASEs, whereas the basolateral route of ATP7A does not traverse any established intermediate endosomal compartments.

Interestingly, we also noticed a pool of ATP7B that constitutively localizes out of TGN in basal and copper-chelated conditions. We determined that this ‘constitutive out-of-TGN ATP7B’ localized at the CRE (marked by 30 min of pulse–chase Tf internalization), suggesting the presence of a copper-independent recycling of ATP7B between the TGN and CREs (Fig. 3C). We could not verify this phenomenon for ATP7A as we could not detect any intermediate trafficking compartment(s) for ATP7A.

### ATP7A and ATP7B exhibit both distinct and similar protein–protein interactions

**Fig. 4.**
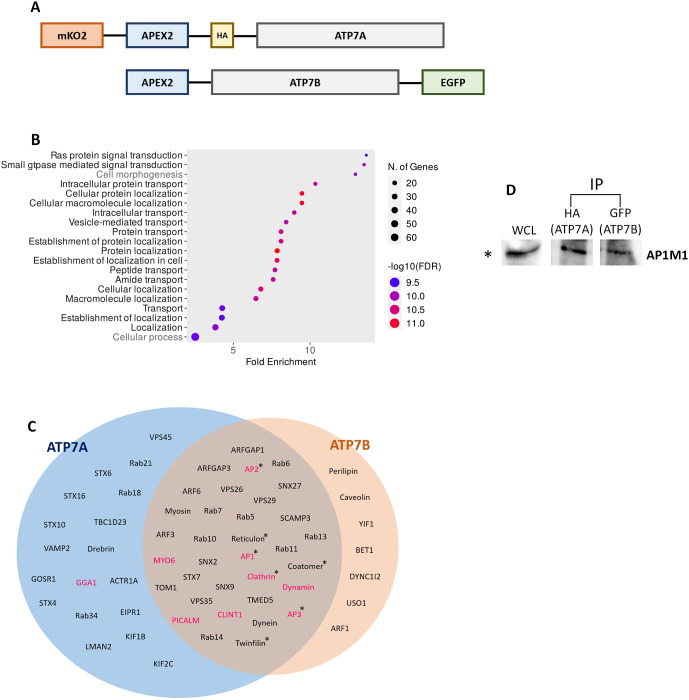
**Interacting partners of ATP7A and ATP7B.** (A) Schematic showing APEX2-tagged ATP7A and ATP7B constructs. (B) Gene Ontology (GO) analysis of 892 proteins identified in the MS data with top 20 groups of biological processes are plotted according to fold enrichment; 18 of the 20 biological process groups are related to protein trafficking (written in black). (C) Venn diagram showing proteins pulled down by APEX2-tagged ATP7A and ATP7B in the proximity biotinylation assay. Trafficking regulators are sorted based on previous reports. Proteins marked in magenta are putative trafficking regulators at TGN or post-TGN compartments. The ‘*’ mark indicates multiple subunits of those proteins are detected. (D) Immuno-pulldown (IP) of mKO2–HA–ATP7A and GFP–ATP7B and probed against AP-1 using rabbit anti-AP1M1 antibody, indicating interaction of AP-1 with both the copper ATPases. WCL, whole-cell lysate (10%). Blot representative of five repeats.

Next, we sought to identify proteins that interact with ATP7B and ATP7A, thus becoming possible candidates to regulate their copper-responsive apical and basolateral trafficking. To this end, we used APEX-2-mediated proximity biotinylation (Rhee et al., 2013); APEX-2 is an engineered peroxidase that can be rapidly induced to tag proteins quickly and efficiently with biotin phenol (BP) and H_2_O_2_ (Lam et al., 2015). APEX-2-tagged ATP7A and ATP7B constructs were generated (Fig. 4A) and stably expressed in MDCK cells. We confirmed that the APEX-2 constructs of ATP7A and ATP7B behaved like the endogenous proteins by localizing at the TGN in basal and copper-chelated conditions and trafficking to their respective plasma membrane domains upon copper treatment (Fig. S4A). Upon biotinylating the interactome of APEX-2-tagged proteins, subsequent pulldown using streptavidin beads and mass spectrometric analysis of the pull-down samples, we found a number of interacting partners for both ATP7A and ATP7B (complete list are in Table S1 for ATP7A, Table S2 for ATP7B). We performed label-free quantification of the pulled proteins (Table S3), and generated a pathway enrichment plot (Fig. 4B), where 18 out of top 20 enriched functional pathways belong to protein localization, trafficking and transport. We further manually clustered the hits based on previous reports, to identify proteins that might be responsible for post-TGN trafficking and recycling (Fig. 4C). Among a spectrum of interacting proteins, we identified various subunits of the adaptor protein AP-1 complex, σ (AP1S1), β (AP1B1), µ (AP1M1 and AP1M2) and γ (AP1G1). In the present study we focused on determining the role of AP-1, as this clathrin adaptor has been previously reported to mediate apico-basolateral cargo sorting at the TGN. We confirmed our mass-spectrometric finding, where we detected µ1A subunit of AP-1 in ATP7A as well as ATP7B complexes (Fig. 4D), by a co-immunoprecipitation assay (Fig. S4B). AP-1 has been shown to interact with ATP7A and ATP7B via the conserved C-terminal di-leucine motifs in non-epithelial models and in neurons (Jain et al., 2015; Yi and Kaler, 2015). The MEDNIK syndrome (an acronym for mental retardation, enteropathy, deafness, neuropathy, ichthyosis, keratodermia), characterized by overlapping symptoms of Wilson and Menkes disease, is caused by a pathogenic variant in the *AP1S1* gene that encodes for the σ1A subunit of AP-1 (Martinelli and Dionisi-Vici, 2014). We studied the regulation of Cu-ATPases by AP-1, as epithelial cells such as MDCK cells express two isoforms of AP-1 (i.e. AP-1A and AP-1B; Ohno et al., 1999). Previously, AP-1A and AP-1B have been shown to play complementary roles in the sorting of basolateral cargos from TGN and endosomes (Gonzalez and Rodriguez-Boulan, 2009; Rodriguez-Boulan et al., 2013; Rodriguez-Boulan and Macara, 2014).

### AP-1 is required for TGN retention of ATP7A and ATP7B

The clathrin adaptor AP-1 has been shown to play a crucial role in the generation and maintenance of plasma membrane protein polarity in epithelial cells. AP-1A and AP-1B isoforms differ in the possession of different medium μ subunits, μ1A and μ1B, respectively (Fölsch et al., 1999; Ohno et al., 1999) and in that they regulate basolateral sorting in different compartments (Rodriguez-Boulan et al., 2013). Although it has been proposed that they have an affinity for different basolateral signals (Bonifacino, 2014), it was initially reported that AP-1 (pan) knockdown results in decrease or loss of polarization for various plasma membrane and recycling basolateral proteins, such as LDLR, TfR, VSVG, ICAM1 and GPRC5A (Gravotta et al., 2007; Kang and Fölsch, 2011), but not of apical proteins, such as gp135, and non-recycling basolateral proteins, such as the Na/K-ATPase (Caceres et al., 2019; Gravotta et al., 2012).

To investigate the roles of AP-1 in trafficking of Cu-ATPases, we first generated MDCK cell lines with double knockout (KO) of AP-1A and AP-1B by knocking out both the medium subunits AP1M1 and AP1M2. MDCK cells with AP-1 (pan) knockout exhibited normal growth and structural polarity that was comparable to wild-type cells. For all the knockouts, we stained the apical membrane with the gp135, the basolateral membrane with ATP1A1 (Na/K ATPase) and the tight junction with the marker ZO-1 (TJP1). They exhibit staining that is in concurrence with proper polarization of the MDCK cells (Fig. S5A, top panel).

**Fig. 5.**
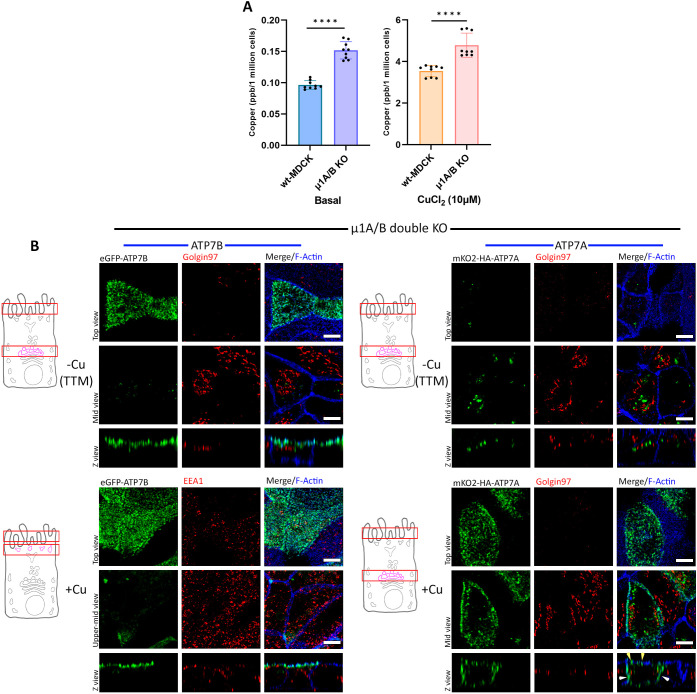
**AP-1 is crucial for TGN retention of ATP7A and ATP7B.** (A) Intracellular copper level in wild-type (wt) and AP-1 KO MDCK cells under basal and copper-treated conditions. AP-1 KO cells show higher copper levels in both conditions [i.e. basal and copper treated cells (10 µM CuCl_2_ for 4 h)] compared to wild-type MDCK cells. Results are mean±s.d (*n*=3). ****P<0.0001 (Mann–Whitney *U*-test/Wilcoxon rank-sum test). (B) Polarized AP-1 KO MDCK cells (µ1A and µ1B double-knockout cells) showing localization of transfected mKO2–HA–ATP7A and eGFP–ATP7B. ATP7B has lost its TGN retention and constitutively localizes to the apical surface, retaining its polarity in copper-depleted as well as copper-treated conditions. ATP7A has also lost its TGN localization and was found to be vesicularized in a copper-chelated environment, but in copper-treated medium it localizes to both the apical (yellow arrowhead) as well as the basolateral surface (white arrowhead), losing its polarity. Images representative of five repeats. Scale bars: 5 µm.

Notably, AP-1 KO cells exhibited higher copper accumulation in basal as well as in copper-treated cells (10 μM CuCl_2_; 4 h), relative to wild-type cells, indicating that AP-1 plays an important role in regulating copper homeostasis in polarized epithelial cells (Fig. 5A). This observation is in concurrence with phenotypes for MEDNIK syndrome, where pathogenic variants of AP-1A lead to an imbalance in copper levels in the cell (Martinelli et al., 2013).

AP-1 (pan) KO had dramatic effects on the localization of both transporters. Under copper-chelated conditions both ATP7A and ATP7B lost their TGN localization, with ATP7B redistributed to the apical surface and ATP7A localized into a vesicular compartment. Upon copper treatment, ATP7B remained constitutively on the apical surface whereas ATP7A was localized on both apical and basolateral surfaces (Fig. 5B). These experiments demonstrate that AP-1 plays a distinct role in the retention of both transporters at the TGN and in the polarized trafficking of ATP7A. A previous study on non-polarized HEK293T cells has shown the involvement of AP-1 in trafficking of ATP7A. Upon knocking down AP-1, the protein lost its TGN localization and constitutively trafficked to the plasma membrane (Yi and Kaler, 2015). Interestingly, our study showed that AP-1 not only regulates TGN retention but also ensures correct trafficking polarity of ATP7A, guiding it to the basolateral membrane.

To test whether AP-1 KO leads to any imbalance in endosomal population, we performed a dextran-uptake assay, which marks the early and recycling endosomal pathway. At three different time points (3 min, 10 min, 60 min) we analyzed the 3D particles per cell to extrapolate the number of dextran-containing vesicles. We found that the distribution of dextran-positive vesicles per cells was comparable between wild-type and AP-1 KO MDCK cells and did not show a biased distribution (Fig. S5B). We also did not observe any apparent change in levels of transcript abundance between ATP7A and ATP7B in copper-treated or copper-chelated conditions in the wild-type, AP-1 (pan KO), AP-1A KO and AP-1B KO MDCK cell lines (Fig. S5C).

### AP-1A and AP-1B cooperation regulates copper-induced TGN exit and sorting of ATP7A and ATP7B

**Fig. 6.**
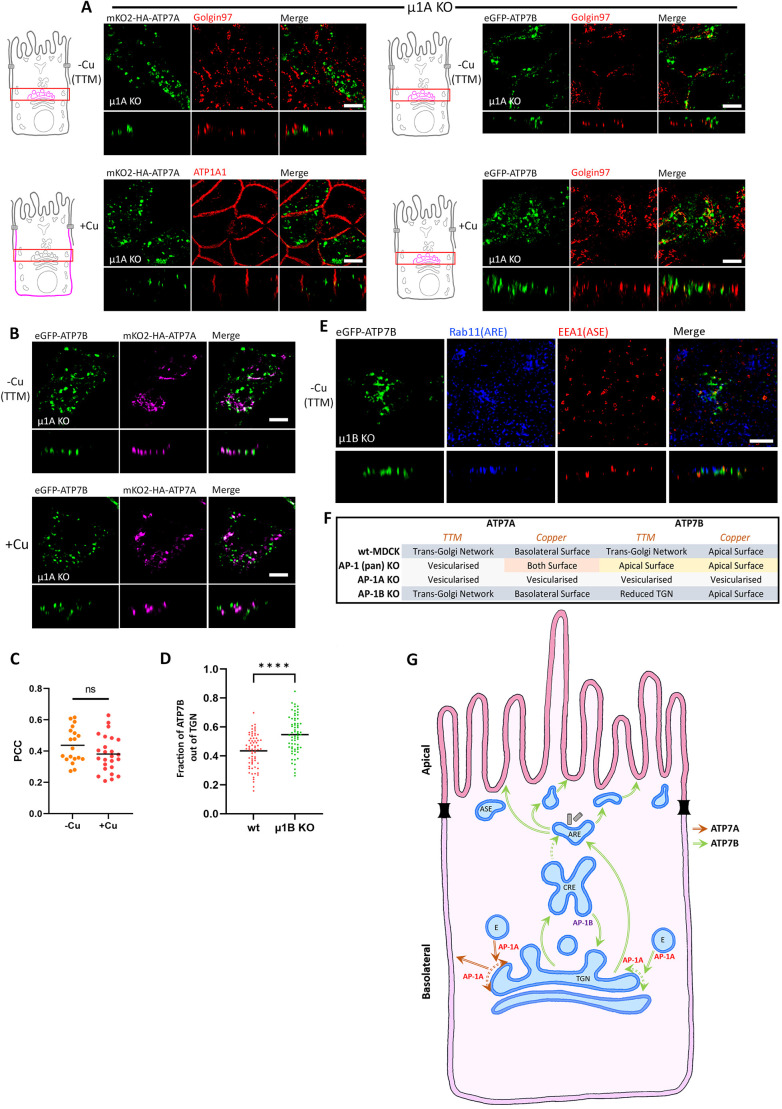
**AP-1A is crucial for TGN retention as well as copper-mediated trafficking.** (A) Images showing localization of ATP7A and ATP7B in polarized AP-1A KO MDCK cells (i.e. µ1A KO cells) in copper-deprived and elevated conditions. Both the Cu-ATPases lost their TGN localization and were found vesicularized in both the copper levels and failed to reach the plasma membrane, which indicates the crucial role of AP-1A in copper-mediated trafficking. (B) Images showing localization of mKO2–HA–ATP7A and eGFP–ATP7B co-transfected in polarized AP-1A KO MDCK cells (i.e. µ1A KO cells) in copper-deprived and elevated conditions. Both ATP7A and ATP7B localize to different endosomal compartments with minimal overlap. (C) Colocalization quantification (Pearson's correlation coefficient) of transfected mKO2–HA–ATP7A with eGFP–ATP7B from B. This shows that there is no effect of copper on the localization of ATP7A and ATP7B in AP-1A KO cells. Line is mean (*n*=−Cu, 19; +Cu, 25). (D) Fraction of transfected eGFP–ATP7B located outside of the TGN marked by golgin-97 (calculated by 1 – Manders' colocalization coefficient) in TTM-treated wild-type (wt) and AP-1B KO MDCK cells. An increased fraction of ATP7B in µ1B KO cells compared to in wild-type cells shows the role of AP-1B in copper-independent recycling. Line is mean (*n*=wt, 68; µ1B KO, 66). (E) Confocal images showing ATP7B in polarized AP-1B KO MDCK cells under copper-chelated conditions. Absence of ATP7B (green) in Rab11-positive (blue) and EEA1-positive (red) compartments suggests lack of anterograde trafficking or spillover from CREs due to regulation by AP-1B in basolateral trafficking at CREs. Images representative of at least three repeats. (F) Tabulated summary of trafficking phenotypes of ATP7A and ATP7B in AP-1A KO, AP-1B KO and AP-1 (pan) KO MDCK cells. (G) Proposed model for copper-independent and copper-dependent trafficking of both the Copper ATPases, i.e., ATP7A and ATP7B, in polarized MDCK cells. Common endosomal stations are marked, like the TGN, CREs, AREs, ASEs and endosomes (labeled with an E). Red arrows mark the trafficking itinerary of ATP7A and the green arrows represents the trafficking path of ATP7B. The dotted green arrow denotes a possible movement of endocytosed cargoes from CREs to the AREs. Dotted double headed arrows denote possible TGN retention by AP-1A. Scale bars: 5 µm.

Next, we dissected the roles of AP-1A and AP-1B in polarized trafficking of ATP7A and ATP7B by generating MDCK cell lines deficient in either μ1A or μ1B, respectively. Proper localization of the apical membrane marker gp135, the basolateral marker ATP1A1 (a Na/K-ATPase subunit) and the tight junction marker ZO-1 confirmed proper polarization of the AP-1A or AP-1B KO MDCK cells (Fig. S5A, middle and bottom panels). In AP-1B KO cells, trafficking of ATP7A and ATP7B was similar to that in wild-type cells. At low copper levels, ATP7A and ATP7B localized at the TGN, and upon copper treatment, they trafficked to the basolateral and apical surfaces, respectively (Fig. S6A). In contrast, AP-1A KO cells displayed a distinct phenotype. Both ATP7A and ATP7B lost their TGN localization but did not traffic to the plasma membrane even upon copper addition. Rather they localized to vesicular compartments in both cases (Fig. 6A), unlike pan-AP-1 knockout, where they trafficked to the cell surface, although in a non-polarized fashion for ATP7A.

Interestingly, in AP-1A KO cells, the vesicularized ATP7A and ATP7B localized at different compartments with minimal overlap. There was no change in this particular localization pattern under the effect of copper (Fig. 6B,C). This observation further substantiates our findings that the decision to follow two distinct itineraries by ATP7A and ATP7B happens at the TGN. Divergence towards their respective apical or basolateral pathways occurs at the TGN and not at any post-TGN compartment(s). We further wanted to determine the nature of the compartments to where ATP7A and ATP7B mislocalize. Interestingly, we did not find appreciable colocalization with Rab GTPases (Rab11, Rab5 and EEA1) or internalized Tf (data not shown). Interestingly dextran uptake for 3 h marked some of these uncharacterized endosomes (Fig. S6B). We found a higher fraction of ATP7A was in dextran-positive compartment compared to that of ATP7B (Fig. S6C). To further investigate the nature of the endosomes, we performed co-staining with LAMP1, which marks the late endosomes and lysosomes. However, we did not find appreciable localization in LAMP1-positive endosomes (Fig. S6D,E), although there were a very few LAMP1 endosomes occupied by both ATP7A and ATP7B.

Our results show that AP-1A functions at the TGN, providing trafficking directionality and TGN retention for both ATP7A and ATP7B. AP-1B does not play a detectable role in the presence of AP-1A, but in the absence of AP-1A, AP-1B facilitates the transport of both transporters to the cell surface, although in a non-polarized fashion for ATP7A. Interestingly, ATP7B but not ATP7A, also localizes at the CREs (marked by 30 min pulse-chase internalization of Tf) in copper-chelated and basal conditions (Fig. 3C), suggesting Cu-independent trafficking of ATP7B from TGN to CRE and, possibly, AP-1B-dependent return from the CREs to the TGN. Consistent with this, AP-1B KO resulted in a significantly higher pool of ATP7B being dispersed out of TGN in copper-chelated conditions (Fig. 6D). AP-1B KO does not facilitate trafficking of ATP7B from CREs to AREs and/or ASEs under copper-chelated conditions (Fig. 6E). By contrast, Perez-Bay and co-workers showed that in MDCK cells with AP-1B knock down, TfR further mislocalizes to AREs deviating from its original plasma membrane–BSE–CRE basolateral itinerary (Perez Bay et al., 2013). This also highlights the differences of AP-1B functioning at the endocytic pathway arising at the plasma membrane versus the late secretory pathway leaving the TGN.

These observations reveal an additional role for AP-1B in the retrograde (copper-independent) route of ATP7B towards the TGN. By contrast, copper-dependent trafficking of ATP7B does not involve its regulation by AP-1B. The phenotypes that we observed for ATP7A and ATP7B in the knockout cells in different copper conditions are summarized in Fig. 6F. Based on our observations in different time scales of copper treatment and the three KO cell lines, we propose a model that illustrates the roles of AP-1 in sorting and trafficking of ATP7A and ATP7B (Fig. 6G).

We confirmed our findings of the KO cells, by performing localization assays for both ATP7A and ATP7B in copper chelated and copper treated conditions in two distinct clonally selected populations of the cells.

### ATP7B mutations suggest additional mechanisms for its TGN retention and subsequent polarized sorting at the TGN

Despite sharing high sequence identity, ATP7B differs completely from ATP7A in its proximal N-terminus domain (amino acids 1–63; Guo et al., 2005), which is critical for TGN exit and proper apical sorting in hepatocytes and WIF-B cells (Hasan et al., 2012). A stretch of nine amino acids (F^37^AFDNVGYE^45^) forms the core minimum motif sufficient for ATP7B apical sorting even in copper-limiting conditions. It contains a N^41^VGY^44^ putative clathrin-binding motif and the N41S hot-spot pathogenic variant, which is responsible for Wilson disease (Braiterman et al., 2009).

**Fig. 7.**
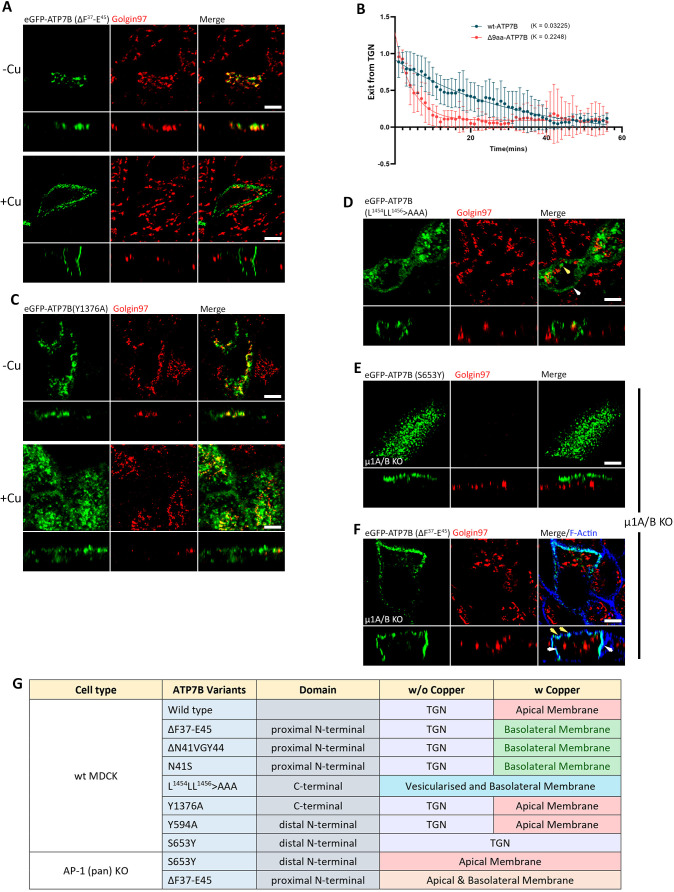
**ATP7B mutants reveal additional regulation for apical polarity.** (A) Images showing the 9-amino-acid (aa) deletion mutant (ΔF^37^-E^45^-ATP7B; Δ9aa) trafficking to basolateral surface in copper-treated conditions. (B) Fitted curve of decreased pixel count of eGFP–ATP7B and Δ9aa mutant in copper-treated conditions, showing dispersion of Cu-ATPases and their exit rate. The higher *k*-value of the Δ9aa mutant shows a faster Golgi exit rate than the wild-type (wt) ATP7B (*N* for ATP7B: 13, Δ9aa mutant: 6). Results are mean±s.d. (C) Confocal image of Y1376A mutant of ATP7B showing wild-type-like phenotype. (D) Image showing that the tri-leucine mutant of ATP7B (i.e. L^1454^LL^1456^>AAA) has lost its TGN localization and localizes to the basolateral surface (white arrowhead) as well as in endosomes (yellow arrowhead). (E) Image of S653Y (a non-Golgi exiting mutant of ATP7B) in AP-1 KO cells (i.e. µ1A/B double knockout cells), which has a constitutive localization on the apical surface of the plasma membrane. (F) Confocal image of ΔF^37^-E^45^ (a basolateral-targeting mutant) in AP-1 (pan) KO cells showing loss of TGN retention as well as in its polarity because of constitutive localization to both the surfaces of the plasma membrane (apical surface marked by yellow arrowhead and basolateral surface marked by white arrowhead). Images representative of three repeats. Scale bars: 5 µm. (G) Tabulated summary of trafficking phenotypes of ATP7B variants in wild-type and AP-1 KO MDCK cells.

We observed that deletion of either the nine residues (ΔF^37^–E^45^) or the core N^41^VGY^44^ motif, and the introduction of the mutant N41S resulted in basolateral sorting of ATP7B under copper stimulation (Fig. 7A; Fig. S7A) and a faster Golgi exit rate of ΔF^37^–E^45^ compared to the wild-type ATP7B (*k*-value 0.2248 versus 0.03225, Fig. 7B; Fig. S7B and Movie 5), which is a phenotype that is reminiscent, albeit milder, of ATP7B losing its TGN retention even in copper-limiting condition in AP-1A KO cells (Fig. 6A, top image, right panel). Similar results were observed in the N^41^VGY^44^ deletion and with the N41S mutation (Fig. S7A).

Given that previous studies on dileucine in the C-terminal of ATP7B have shown its AP-1 interaction and its involvement in somatodendritic polarity (Jain et al., 2015), we screened the cytosolic domains of ATP7B for the presence of additional clathrin-interacting motifs. Mutations on two YxxФ motifs on the C-terminal (Y^1376^>A; Fig. 7C) or the N-term domain (Y^594^>A, Fig. S7C) did not affect the trafficking phenotype. Mutating the tri-leucine motif (L^1454^–L^1456^) essential for retrograde trafficking (Braiterman et al., 2011) (L^1454^LL^1456^>AAA) resulted in loss of TGN localization, even in TTM-treated cells (Fig. 7D), similar to the phenotype observed in AP-1A KO cells.

A copper-insensitive Wilson disease-causing pathogenic variant, ATP7B-S653Y (Thompson et al., 2007), did not exit the TGN in wild-type MDCK cells (Fig. S7D), but trafficked to the apical plasma membrane in a copper-independent manner in pan-AP-1 KO MDCK cells (Fig. 7E). By contrast, the ATP7B-ΔF^37^-E^45^ mutant in pan-AP-1 KO MDCK cells also lost its TGN retention in copper-limiting conditions, which is preserved in wild-type MDCK cells. Interestingly, ATP7B-ΔF^37^-E^45^ loses its trafficking polarity to both the membrane surfaces, contrary to what occurs in the wild-type, where it was localized only to the basolateral surface (Fig. 7F). These findings substantiate the regulatory role of AP-1 in the TGN retention of the Cu-ATPases. The phenotypes that we observed for ATP7B mutants in the wild-type and knockout cells in different copper conditions are summarized in Fig. 7G.

To summarize, our studies on ATP7B mutants expressed in wild-type and AP-1 KO cells suggest a strong role for AP-1 in TGN retention, but the directionality of ATP7B depends on AP-1 in synergy with other regulatory signals or regulators. Mutating putative clathrin-binding motifs in ATP7B did not result in the protein following the exact trafficking itinerary observed in AP-1 KO cells. This indicates the presence of a multi-tier regulatory mechanism for copper-dependent trafficking of ATP7B. Further studies on this regulation could be fascinating as polarized epithelial cells can reveal new insights into stepwise control of polarized trafficking.

Our study sheds light on the molecular mechanism of the overlap in phenotypes of the copper metabolism diseases Wilson and Menkes disease with MEDNIK syndrome. Our study also unravels the presence of a default trafficking pathway that directs proteins to the basolateral membrane.

## DISCUSSION

Copper levels in a mammalian cell are primarily regulated by the homologous P-type copper-ATPases ATP7A and ATP7B. We determined the underlying mechanism of basolateral versus apical trafficking of ATP7A and ATP7B, respectively, in response to elevated copper in a model of polarized epithelia.

ATP7A and ATP7B, as two distinct copper-transporting ATPases, only appear in teleosts. In invertebrates, a single copper-ATPase was possibly responsible for copper transport in the biosynthetic pathway, as well as for exporting excess copper. With the evolution of two copper ATPases, the functional responsibility was divided with respect to (1) copper uptake from the alimentary canal to the bloodstream for systemic copper distribution and (2) biliary copper export for detoxification. In parallel to this functional specialization, the trafficking polarity of the two copper ATPases came into existence, with the copper-distributing ATPase ATP7A, which transports copper into the bloodstream, attaining basolateral trafficking polarity, and the copper-detoxifying ATPase ATP7B gaining the apical or luminal trafficking polarity. We asked what could be the possible mechanism that regulates the opposite trafficking polarity of such evolutionary and functionally similar membrane transporters.

Epithelial cells contain two isoforms of AP-1 clathrin adaptor complexes. AP-1A is ubiquitously expressed and regulates transport between the TGN and endosomes. AP-1B is expressed only in epithelia and mediates the polarized targeting of membrane proteins to the basolateral surface (Fölsch et al., 2003). A study from Bonifacino's group was the first to identify a novel member of the adaptor medium chain family, μ1B, which is closely related to μ1A and shares a 79% amino acid sequence identity (Fölsch et al., 1999; Ohno et al., 1999). In a review that summarizes the developments in the field of clathrin over the last 40 years, Robinson mentions that vertebrates were the first organisms where epithelial cell-specific isoforms of the AP-1 medium (µ) subunit emerged, which contributes to basolateral sorting (Robinson, 2015). We hypothesize that the divergence of homologous membrane proteins towards the apical and basolateral membrane of polarized epithelia coincided with appearance of the two isoforms of AP-1 (i.e. AP-1A and AP-1B).

Using a mass-spectrometric approach, we identified multiple partners that could regulate the apico-basolateral trafficking polarity of ATP7A and ATP7B. A pool of hits emerged that constituted subunits of adaptor proteins (APs), specifically, AP-1. The AP complexes are hetero-tetrameric protein complexes that mediate intracellular membrane trafficking at endocytic and secretory pathways. There are five different AP complexes; AP-1, AP-2 and AP-3 are clathrin-associated complexes, whereas AP-4 and AP-5 are not (Park and Guo, 2014). Mutations on AP subunits have been implicated in a variety of inherited disorders, including MEDNIK syndrome, Hermansky–Pudlak syndrome, Fried syndrome and hereditary spastic paraplegia (HSP) (Abdollahpour et al., 2015; Alsaif et al., 2019; Borck et al., 2008; Dell'Angelica et al., 1999). AP-1 is a highly conserved clathrin adaptor that functions primarily at the TGN, and also at endosomes and lysosomes (Zysnarski et al., 2019).

Growing evidence has implicated the involvement of AP-1 in disorders of copper metabolism. MEDNIK syndrome is caused by a pathogenic variant in the AP-1 sigma (σ) subunit, and shares multiple symptoms and cellular phenotypes with Wilson and Menkes disease, hence indicating the possible involvement of AP-1 in regulation of ATP7A and ATP7B (Martinelli and Dionisi-Vici, 2014). Recently, Alsaif et al. identified a pathogenic variant in AP1B1, which encodes the large β subunit of the AP-1 complex, that causes MEDNIK-like syndrome (Alsaif et al., 2019). The affected individuals manifested abnormal copper metabolism, evidenced by low plasma copper and ceruloplasmin, but normal levels of hepatic copper. We also detected higher copper levels in AP-1 KO cells. Our mass-spectrometric data and these previous genetic and clinical reports prompted us to conduct an in-depth study of the regulation of the Wilson and Menkes disease-associated proteins by the adaptor protein AP-1.

Although it has been well established that upon copper treatment in polarized epithelial cells, ATP7A traffics to the basolateral end and ATP7B towards the apical end, the trafficking itinerary in these cells had not been previously determined. We found that ATP7A follows a direct route from the TGN that bypasses any of the known sorting endosomal compartments implicated in basolateral trafficking (e.g. CREs, basolateral sorting or the basolateral endosomes). By contrast, ATP7B traverses through a more elaborate itinerary that involves CREs, ASEs and AREs. Interestingly, our findings are in contradiction to those of Nyasae et al. (2014) in WIF-B cells, where ATP7B localizes at the BSE before being transported to the apical membrane. Differences in cell types and lineages might be responsible for this contrasting finding.

We discovered a pool of ATP7B (and not ATP7A) that exhibits copper-independent post-TGN and CRE localization. Interestingly, this pool of copper-independent ATP7B is distinct from the more abundant copper-exporting ATP7B pool that traverses through the AREs and ASEs. We hypothesize that these compartments might serve as a storage site for copper that might be eventually utilized in situations of copper deficiency. However, further investigation is warranted to establish this model. We found that AP-1B regulates this distribution of ATP7B between TGN and the CREs.

To summarize, we propose a model that illustrates the roles of AP-1 in sorting and trafficking of the homologous copper-transporting ATPases ATP7A and ATP7B. AP-1A functions at the TGN providing directionality and possibly TGN retention for both ATP7A and ATP7B. However, in the present scope of the study, it is difficult to pinpoint whether in absence AP-1A, the ATPases lose their ability to be retained at the TGN or their constitutive recycling to the TGN from the post-TGN compartment is compromised. In absence of AP-1A, AP-1B works at the TGN-retention checkpoint for both the Cu-ATPases. This finding agrees with those of Gravotta et al. (2012), which demonstrates a critical role of the ubiquitous AP-1A in basolateral sorting. Knockdown of AP-1A causes mis-sorting of basolateral proteins in MDCK cells, but only after knockdown of AP-1B, suggesting that AP-1B can compensate for the lack of AP-1A.

In concurrence with our findings, Gravotta et al. (2012) also suggest that knockdown of AP-1B promotes ‘spillover’ of basolaterally trafficking proteins into AREs from CREs (the site of function of AP-1B), suggesting complementary roles for AP-1A and AP-1B adaptors in basolateral sorting. We found that in absence of both AP-1A and AP-1B, ATP7A loses its trafficking polarity and localizes at both membranes in response to copper. By contrast, ATP7B loses its TGN retention capacity but retains trafficking polarity, which now becomes independent of intracellular copper concentration.

Our findings suggest that beside regulating sorting at the TGN, AP-1 might play a role in exit of cargoes from endosomal compartments. Delevoye et al. demonstrated that AP-1, and its interacting motor KIF13A, cooperate to generate recycling endosomal domains that are specified for communication with melanosomes. AP-1 and the KIF cooperate in cargo sorting and in positioning endosomes at the cell periphery near melanosomes, permitting the formation of interorganellar tubular connections (Delevoye et al., 2009).

Besides adaptor proteins, we have identified the role of the N-terminus of the Cu-ATPases in directing them to apical or basolateral membranes upon copper treatment. Our own, as well as studies, from other groups have demonstrated that copper binding to the N-terminal domain and associated structural changes do influence sorting and TGN exit signals. Hasan et al. has shown that regulatory kinase-mediated phosphorylation at the serine stretch (S^340^–S^343^) between MBD3 and MBD4 regulates exit of ATP7B from the TGN in response to copper. Mutating S340 and/or S341 in the N-terminal domain of ATP7B individually or together to alanine, glycine, threonine or aspartate residues shifts the steady-state localization of ATP7B to vesicles, independently of copper levels. Furthermore, this region also is responsible for copper-dependent interaction between the N-terminus and the nucleotide-binding domain (Hasan et al., 2012).

Braiterman and co-workers narrowed down the apical targeting sequence of ATP7B to nine amino acids, F^37^–E^45^, which constitutes an essential apical-targeting determinant for ATP7B in elevated copper and in TGN retention of the protein under low-copper conditions (Braiterman et al., 2009). In this present study, we have utilized multiple Wilson disease-causing pathogenic variants as well as the ΔF^37^–E^45^ deletion to characterize possible associated changes at the N-terminus that influence TGN exit and subsequent trafficking of ATP7B. To summarize, we hypothesize a model where there is a concerted interplay between the APs and the N-terminus of the Cu-ATPases to determine their apico-basolateral polarity. Additionally, it would be interesting to characterize the role of other identified interacting proteins identified in the mass spectroscopy data (e.g. AP-3 and ARF proteins) in regulating apical versus basolateral trafficking of these homologous ATPases.

The importance of our study lies in establishing the roles and understanding the mechanism of the clathrin adaptor protein AP-1 in copper metabolism by regulating the trafficking dynamics of the Wilson and Menkes disease-associated proteins. Many patients exhibit a spectrum of Wilson disease-like clinical symptoms, but upon gene sequencing, *ATP7B* or copper-metabolism pathways genes reveal no mutations. It will be important to screen AP-1A and AP-1B in those patients, as that might reveal novel pathogenic variants thus enabling better disease diagnosis.

## MATERIALS AND METHODS

### Plasmids and antibodies

The eGFP–ATP7B construct was available in the lab previously (Das et al., 2020). HA–ATP7A was a kind gift from Michael Petris (University of Missouri, Columbia, MO, USA) and Svetlana Lutsenko (Johns Hopkins University, Baltimore, MD, USA). The mKO2 fluorescent tag was added to the N-terminal of ATP7A with the final construct denoted as mKO2–HA–ATP7A. APEX2–ATP7B–eGFP and APEX2–mKO2–HA–ATP7A constructs were made from the existing plasmid using a NEBuilder HiFi DNA Assembly kit (NEB #E2621). Mutations on eGFP–ATP7B were prepared following the Q5 Site-Directed Mutagenesis Kit (NEB #E0554) protocol. Primer details are in Table S4. Plasmid isolation was performed using a Macherey Nagel plasmid isolation kit (#MN740490).

The following antibodies were used in this study: mouse anti-golgin-97 (Thermo Fisher Scientific #A21270) at 1:400; rabbit anti-golgin-97 [Cell Signaling Technology (CST) #13192] at 1:500; mouse anti-p230 (BDBiosciences #611280) at 1:1000; mouse anti-ATP1A1 (Abcam #ab7671) at 1:300; mouse anti-gp135 (DSHB #3F2/D8) at 1:400; mouse anti-EEA1 (BD Transduction #610456) at 1:250; rabbit anti-Rab11 (Thermo Fisher Scientific #715300) at 1:300; rabbit anti-LAMP1 (Affinity Biosciences #DF4806) at 1:400; rabbit anti-AP1M1 (Sigma #SAB1301057) at 1:1500; rabbit anti-AP1M2 (Sigma #SAB2105800) at 1:1500; rabbit anti-α tubulin (Affinity Biosciences #AF7010) at 1:15000; rabbit anti-ZO-1 (Invitrogen #40-2200) at 1:300; mouse anti-HA (Biolegend #901501) at 1:100; mouse anti-GFP (DSHB #DSHB-GFP-12A6) at 1:100; anti-mouse-IgG conjugated to Alexa Fluor 488 (Invitrogen #A11029) at 1:800; anti-mouse-IgG conjugated to Alexa Fluor 555 (Invitrogen #A32773) at 1:800, anti-rabbit-IgG conjugated to Alexa Fluor 568 (Invitrogen #A11011) at 1:800; anti-mouse-IgG conjugated to Alexa Fluor 647 (Invitrogen #32787) at 1:800; and anti-rabbit-IgG conjugated to HRP (CST #7074S) at 1:5000. To mark the TGN, golgin-97 or p230 (golgin-245) were used. We confirmed that these two proteins localize at the same TGN compartment by co-staining and subsequent imaging under a confocal microscope (Fig. S7E)

### Cell lines and cell culture

MDCK cells were from our laboratory and have been described before in our recent studies (Das et al., 2022) and tested negative for any contamination. MDCK cells were grown and maintained in medium consisting of DMEM (Gibco #11995056), supplemented with 10% FBS (Gibco #26140079) and 1× Pen-Strep (Gibco #15140122) at 37°C and 5% CO_2_. For monolayer polarization, 3×10^5^ cells were plated in 0.4 µm, 12 mm inserts (Corning #3401) and grown for 3–4 days. For transfection, electroporation was performed using a Nucleofector 2b device (Lonza #AAB-1001) and Amaxa Kit V (Lonza #VCA-1003) Program T-023.

For copper treatment, 50 μM of CuCl_2_ (SRL #92315) was used for 2 h, unless otherwise mentioned. For mimicking copper-deprived conditions, cells were treated with 50 μM BCS (SRL #28242) or 25 μM TTM (Sigma #323446) for 2 h.

For the establishment of stable cell lines, respective plasmids were transfected, and cells were maintained in selection medium containing 1 μg/ml puromycin (Gibco #A1113803) for 48 h. After that cells were grown in fresh medium, and visible colonies were isolated using cloning cylinders and subjected to fluorescence-activated cell sorting (FACS), only selecting low to medium-expression cells.

### Immunofluorescence and microscopy

After desired treatments, cells were washed twice with ice-cold PBS, then fixed with 2% PFA for 20 min. Cells are washed with PBS followed by 20 min incubation in 50 mM ammonium chloride solution. Next, the cells were washed with PBS, and blocking and permeabilization were performed in 1% bovine serum albumin (BSA, SRL #85171) in PBSS (0.075% saponin in PBS) for 20 min. Primary antibody incubation was performed for 2 h at room temperature (RT) followed by three PBSS washes. After that, incubation with the respective secondary antibodies was performed for 1 h followed by three PBSS washes. The membrane was mounted with the ProLong Gold Antifade Reagent (CST #9071). All images were acquired with a Leica SP8 confocal platform using a oil immersion 63× objective (NA 1.4) and deconvolved using Leica Lightning software. All the images were captured at *z*-interval of 0.2 µm.

For determining the Golgi-exit rate, cells were transfected with eGFP–ATP7B or mKO2–HA–ATP7A and plated in glass bottom dishes or 0.4 µm, 12 mm inserts. Before imaging, phenol-containing medium was replaced with imaging medium [DMEM without Phenol Red (Gibco #31053028), 20 mM HEPES (Gibco #15630106), 1 mM Trolox (Sigma #238813) and 1% FBS]. 50 µM CuCl_2_ was added, and cells were imaged at an interval of 60 s for 1 h.

### Image analysis

For calculating the Golgi-exit kinetics, a uniform mean-intensity range was used in all the slices by resetting the minimum and maximum intensities. From the histogram, pixels having more than 95% of the maximum intensity value (in this case 65653 as a 16-bit image is used) were considered and counted as Golgi in basal condition. Upon copper treatment, the disappearance of those pixels was considered as Golgi exit. The decrease in pixel count was plotted against time. All images were processed and analyzed using ImageJ software. The ImageJ macro code for the analysis is available at https://github.com/saps018/7A-7B-.

For colocalization calculation, the JaCoP plugin (https://biop.epfl.ch/Fiji-Update/) was used. Manders' or Pearson's colocalization coefficients were calculated from manually drawn regiosn of interest (ROIs). For particle analysis, the ImageJ 3D Objects Counter plugin was used. The same number of *z*-slices were considered for all images. Graphs were plotted using GraphPad Prism (version 9.4).

### Uptake assays

For 633–Tf (Invitrogen #T23362) uptake assays, cells were starved for 60 min at 37°C in HBSS containing 20 mM HEPES (standard buffer) and incubated for 60 min at 4°C with 15 μg/ml 633–Tf in 1% BSA in standard buffer. After that, the temperature was shifted to 37°C and, after the specified time, cells were immediately rinsed with ice-cold HBSS and fixed with ice-cold 2% PFA in PBS. After quenching PFA with 50 mM NH_4_Cl (dissolved in PBS) for 15 min, cells were permeabilized with PBSS for 5 min. Following this, cells were incubated with corresponding primary antibodies for 2 h and then with secondary antibodies again for 2 h in 1% BSA in PBSS and washed three times with PBSS after each incubation.

To label BSEs and CREs, Tf–633 was added to the basolateral side of the cells growing on the 12-mm inserts , for 5 min and 30 min, respectively, at 37°C (15 μg/ml in 1% BSA standard buffer).

To label ASEs, cells were exposed to 633–WGA (Invitrogen #W21404) from the apical side for 5 min at 37°C (25 μg/ml in 1% BSA standard buffer). Excess WGA was stripped by washing three times with 100 mM N-acetyl-D-glucosamine (Sigma #SLCJ4809) in HBSS for 10 min at 4°C and then cells were fixed and stained with respective antibodies.

To label endocytic endosomes, 647–dextran (Invitrogen #D22914) was internalized from the basolateral side (40 μg/ml in 1% BSA standard buffer). Cells were fixed after specified time and mounted immediately on glass slides and imaged the next day.

### Quantitative real-time PCR

MDCK cells were seeded in 60 mm culture dishes. After cells reached confluency, medium was discarded, and cells were harvested using 1 ml TRIzol reagent (Thermo #15596018). All the downstream processes were performed according to the manufacturer's protocol. Briefly, after 5 min incubation, 0.2 ml chloroform (SRL #96764) per 1 ml of TRIzol was added to the harvested cells and mixed by mild shaking. After that, the mixture was centrifuged for 15 min at 12,000 ***g*** at 4°C. 200 µl of the aqueous phase was collected and 500 µl isopropanol (SRL #38445) was added to it and incubated for 10 min followed by centrifugation at 12,000 ***g*** for 10 min at 4°C for precipitation of RNA. The RNA pellet was washed twice with 500 µl of 75% ethanol followed by centrifugation at 12,000 ***g*** for 5 min at 4°C. After final wash, the supernatant was discarded completely, and the RNA pellet was air dried and was reconstituted in 20 µl DEPC-treated nuclease-free water. RNA samples were run on gel to check the quality of RNA and subsequently cDNA was synthesized using Verso cDNA Synthesis Kit (Thermo Fisher Scientific #AB1453A) according to the manufacturer's protocol, where equal amounts of RNA was added to each reaction.

For real-time PCR, SYBR green PCR master mix (BioRad #125121) was used and the reaction was performed in BioRad CFX-96 real-time system. The relative transcript level of all the genes were normalized against wild-type MDCK cells and the *GAPDH* gene was taken as endogenous control. The experiment was performed as per MIQE guidelines. Details of primers are listed in Table S5.

### Intracellular copper measurement

An equal number of cells (wild-type MDCK or AP-1 KO MDCK cells) were seeded in 60 mm culture dishes. After they reached confluency, cells were treated with either TTM, BCS, CuCl_2_ or left in basal copper conditions. Cells were then washed with ice-cold PBS several times (5–6 times) and were harvested in centrifuge tubes (400 ***g*** for 2 min). Next, cells were counted, and an equal number of cells were digested in 100 μl of nitric acid (Merck #1.00441.1000) overnight at 95°C. Copper standards were prepared from 23 Element standard (Reagecon #ICP23A20). To bring the final concentration of nitric acid ≤2%, digested samples were diluted in 5 ml MilliQ water (Millipore) and analyzed using an Xseries 2 ICP-MS machine (Thermo Fisher Scientific). Values were plotted using GraphPad Prism.

### Proximity biotinylation

Cells stably expressing either APEX2–mKO2–HA–ATP7A or APEX2–ATP7B–eGFP were plated in 0.4 µm, 24 mm inserts and cultured until they reached confluency. 2 mM biotinyl tyramide (Sigma #SML2135)-containing medium was added 30 min prior to copper treatment. Cells were washed three times in PBS^++^ (with Ca^2+^/Mg^2+^). The peroxidase reaction was performed for 1 min using 0.5 mM H_2_O_2_ followed by quenching in quencher solution (PBS^++^ with 10 mM sodium ascorbate, 5 mM Trolox and 10 mM sodium azide). Subsequently, cells were washed with quencher solution twice. Next, cells were scrapped in RIPA lysis buffer [10 mM Tris-HCl pH 8.0, 1 mM EDTA, 0.5 mM EGTA, 1.0% Triton X-100, 0.1% sodium deoxycholate, 0.1% SDS, 150 mM NaCl, 1× protease inhibitor cocktail (Sigma #P8340), 1 mM PMSF] and flash frozen. Samples were thawed and sonicated with a probe sonicator (4 pulses, 30 s, and 70 mA). Lysates were clarified by centrifugation at 18,000 ***g*** for 10 min at 4°C.

For the isolation of biotinylated proteins, streptavidin-coated magnetic beads (Genscript #L00936) were prepared by washing twice with RIPA lysis buffer. Clarified lysates from the above step were added to the beads and incubated at room temperature for 2 h with gentle mixing. For each sample 100 μL of bead slurry was used. Streptavidin beads were then washed three times with RIPA lysis buffer. Biotinylated proteins were eluted in 1.5× NuPAGE LDS loading buffer (Invitrogen #NP0007) containing 20 mM DTT (SRL #17315) and 2 mM biotin (SRL #18888) by heating at 95°C for 10 min.

### In-gel digestion and extraction

Biotinylated proteins eluted from magnetic streptavidin beads were separated on a NuPAGE Novex Bis-Tris 4–12% gel run for 10 min at 120 V. Lanes for each sample were manually cut into 1×1 mm cubes. Gel bands were de-stained with 300–800 µl of 70% 50 mM ammonium bicarbonate and 30% acetonitrile for 30–60 min followed by dehydration with acetonitrile. Dehydrated gel bands were swelled with 150 µl of 10 mM DTT in 50 mM ammonium bicarbonate for 45 min at 56°C. The DTT solution was subsequently removed and replaced with 150 µl of 55 mM iodoacetamide in 50 mM ammonium bicarbonate and incubated in the dark for 30 min. The iodoacetamide solution was removed and bands were dehydrated with acetonitrile. Approximately 30–50 µl of 15 ng/µl sequencing grade trypsin (Promega) was added to each sample and digestion was completed overnight at 37°C. After the overnight digestion, excess trypsin solution was removed from each sample and bands were incubated with 150 µl of extraction solution (5% formic acid, 30% acetonitrile). The extraction solution was collected after 10 min, and this process was repeated once. The final peptide extraction was completed by incubating gel bands with 100% acetonitrile for 5 min. Extracted peptides were dried to completeness in a vacuum concentrator. For desalting, samples were reconstituted in 20 µl of 0.1% formic acid and loaded onto C18 StageTips. The tips were washed twice with 50 µl of 0.1% formic acid, and peptides were eluted with 50% acetonitrile with 0.1% formic acid and then dried in a vacuum concentrator.

### Liquid chromatography and mass spectrometry

Samples were analyzed on a Q-Exactive HF mass spectrometer coupled with nanoflow HPLC System (Easy-nLC 1200, Thermo Fisher Scientific). The sample was loaded on to Easy Spray Column PepMap™ RSLC C18 (3 μm, 100 A^0^ 75 μm×15 cm). Samples were eluted with a 60 min gradient as detailed in Table S6.

The MS was performed with a Orbitrap analyzer at the resolving power of 60,000 at *m*/*z* 200. The scan range selected was 400–1650 *m*/*z*. Tandem MS (MS/MS) was carried out in HCD (normalized collision energy, 28%) mode with resolving power of 15,000 at *m*/*z* 200. The top 10 ions were taken for MS/MS. The lock mass option was enabled for accurate mass measurements.

Proteins and peptides were identified and quantified using the MaxQuant software package (version 2.1.0.0; Cox et al., 2011; Cox and Mann, 2008). Data were searched against the UniProt canine database, which contains 59,102 entries. A fixed modification of carbamidomethylation of cysteine and variable modifications of N-terminal protein acetylation, oxidation of methionine, and biotin-phenol on tyrosine were searched. The enzyme specificity was set to trypsin allowing cleavages N-terminal to proline and a maximum of two missed cleavages was utilized for searching. The maximum precursor ion charge state was set to 6. The precursor mass tolerance was set to 20 ppm for the first search where initial mass recalibration is completed and 4.5 ppm for the main search. The MS/MS tolerance was set to 20 ppm and the top MS/MS peaks per 100 Da was set to 12. The peptide and protein false discovery rates were set to 0.01 and the minimum peptide length was set to 6. Then the results were analyzed in Perseus (version 2.0.10.0). For gene enrichment study, the ShinyGO graphical gene-set enrichment tool was used (Ge et al., 2020). The clustering was based on Gene Ontology (GO) Biological Process (Aleksander et al., 2023; Ashburner et al., 2000; Thomas et al., 2022). The false discovery rate (FDR) cutoff was 0.05 and minimum 15 genes was considered for a pathway.

### Co-immunoprecipitation and immunoblot analysis

MDCK cells expressing either mKO2–HA–ATP7A or eGFP–ATP7B were seeded in equivalent numbers in 60 mm culture dishes and grown till they reached 60 to 70% confluency. On the day of the experiment, cells were treated with 50 μM CuCl_2_ for 15 min, and subsequently lysed using a syringe in non-degrading lysis buffer (50 mM Tris-HCl pH 7.5, 150 mM NaCl, 0.1% CHAPs, 1 mM EDTA supplemented with 1× PIC, 1× PhosSTOP and 1 mM Na_3_VO_4_) for nearly up to 1 h. Lysates were centrifuged at 10,600 ***g*** at 4°C for 5 min. The supernatant (i.e. the crude cell lysate) was then subjected to a Bradford assay to measure the total protein concentration. Simultaneously, Protein-G-coated MagBeads (Genscript #L00274) were equilibrated with binding/wash buffer (20 mM Na_2_HPO_4_, 150 mM NaCl, pH 7.0) and incubated with the target IgG at room temperature while mixing on a rotator for 60 min. Cross-linking of mouse anti-HA or mouse anti-GFP antibodies (1 µg of antibody per 100 µg of protein) to the MagBeads was done using freshly prepared cross-linking solution (20 mM dimethyl pimelimidate dihydrochloride in 0.2 M triethanolamine, pH 8.2) for 30 min at room temperature using rotational mixing. The cross-linking reaction was stopped using 50 mM Tris-HCl pH 7.5 for 15 min at room temperature using rotational mixing post which the cross-linked beads were washed three times with PBS, pH 7.4. An equal amount of protein extracts (100 μg) was added to the cross-linked MagBeads for overnight binding in a rotator at 4°C. After three washes of the protein-captured beads with PBS, 1× NuPAGE LDS loading buffer was added and heated at 95°C for 10 min to denature the proteins and separate them from the beads. The proteins were separated in SDS-PAGE and transferred onto PVDF membranes. After blocking with 3% BSA in TBST (Tris-buffered saline with 0.1% Tween 20) for 2 h, the membranes were probed with primary antibodies against AP1M1 at 4°C overnight, followed by secondary antibody conjugated to HRP. Then, the membranes were developed (Bio-Rad #1705062) and visualized using ChemiDoc (Bio-Rad).

### Generation of AP1 knockout MDCK-II cell lines

For AP-1 subunit genetic knockout, a CRISPR-Cas9-mediated non-homologous end joining (NHEJ) strategy was implemented. Briefly, guide RNA selection was determined by picking the first common exon based on the NCBI Gene curated transcript database for each gene. The first shared exon of all known transcripts was then used as the ‘target’ for guide selection in the CHOPCHOP guide selection tool (http://chopchop.cbu.uib.no). The highest scoring 19 bp guide was then synthesized with an additional guanidine nucleotide at the 5′ end of the guide sequence for efficient U6 promoter transcription, and with BbsI sticky-ends for cloning into the BbsI linearized pX459v2 (Ran et al., 2013). The guide sequences used were: AP1M1, 5′-TCATCTGCCGGAATTACCG-3′; AP1M2, 5′-CATGCCTCTGCTCATGCAG-3′.

A total of 4×10^6^ MDCK cells were transfected with 5 µg of the pX459v2-AP1M1 or pX459v2-AP1M2 or both the plasmids with the Lonza AMAXA 2B nucleofection system. Freshly transfected cells were plated in 150-mm dishes (30,000 and 1000 cells per dish). After 24 h, 1 µg/ml puromycin was added, followed by a change to fresh medium after 48 h. Cells were left to grow for an additional 7 to 10 days to expand the single clones, and subsequently single colonies were picked up using glass cloning cylinders by trypsinizing them and plating the clones to a 96-well plate. These clones were sequence verified to select knockouts for the desired gene. Sequencing was performed on PCR products obtained from cDNA of the knockout clones. The following primers were used for PCR amplification and subsequent reverse primers are used for sequencing. AP1M1 Seq Forward Primer, 5-′AGTGGAGGCGAACTGTCG-3′, AP1M1 Seq Reverse Primer, 5′-CTTGCTGTCGGTGGTCTGG-3′; AP1M2 Seq Forward Primer. 5′-CGCCTCGGCTGTCTTCATC-3′, AP1M2 Seq Reverse Primer, 5′-GATTTGCCAGTCACCAGCTTG-3′. Sequence alignment is shown in Fig. S7F.

We also verified the protein expression of these knockouts. In AP-1 (pan) KO MDCK cells, we recorded negligible to absolutely no expression of AP1M1 and AP1M2. Also, in AP-1A KO MDCK cells, we verified the absence of AP1M1 (Fig. S7G). However, we could not verify the level of AP1M2 in AP-1B KO MDCK cells due to poor specificity and cross reactivity of AP1M2 antibody available in the laboratory. To ensure equal loading, the same blots were stripped and re-probed with anti-α-tubulin antibody. The stripping solution was 62 mM Tris-HCl pH 6.8, 2% SDS and 100 mM 2-mercaptoethanol, incubated at 56°C for 20 min.

All the plasmids and constructs are available on request to the authors.

### Statistical analysis

All the analyses were undertaken using GraphPad Prism (version 9.4), where nonparametric Mann–Whitney *U*-test/Wilcoxon rank-sum test was performed. *P<0.05, **P<0.01, ***P<0.001, ****P<0.0001, ns; not significant.

## Supplementary Material

10.1242/joces.261258_sup1 Supplementary information

Table S1.

Table S2.

Table S3.
