# Supplementary material for: Regulation of the apico-basolateral trafficking polarity of the homologous copper-ATPases ATP7A and ATP7B
Source: J Cell Sci. 2023 Nov 30;137(5):jcs261258. doi: 10.1242/jcs.261258 (PMC10729821; doi:10.1242/jcs.261258)
Supplement: Supplementary information [file joces-137-261258-s1.pdf]

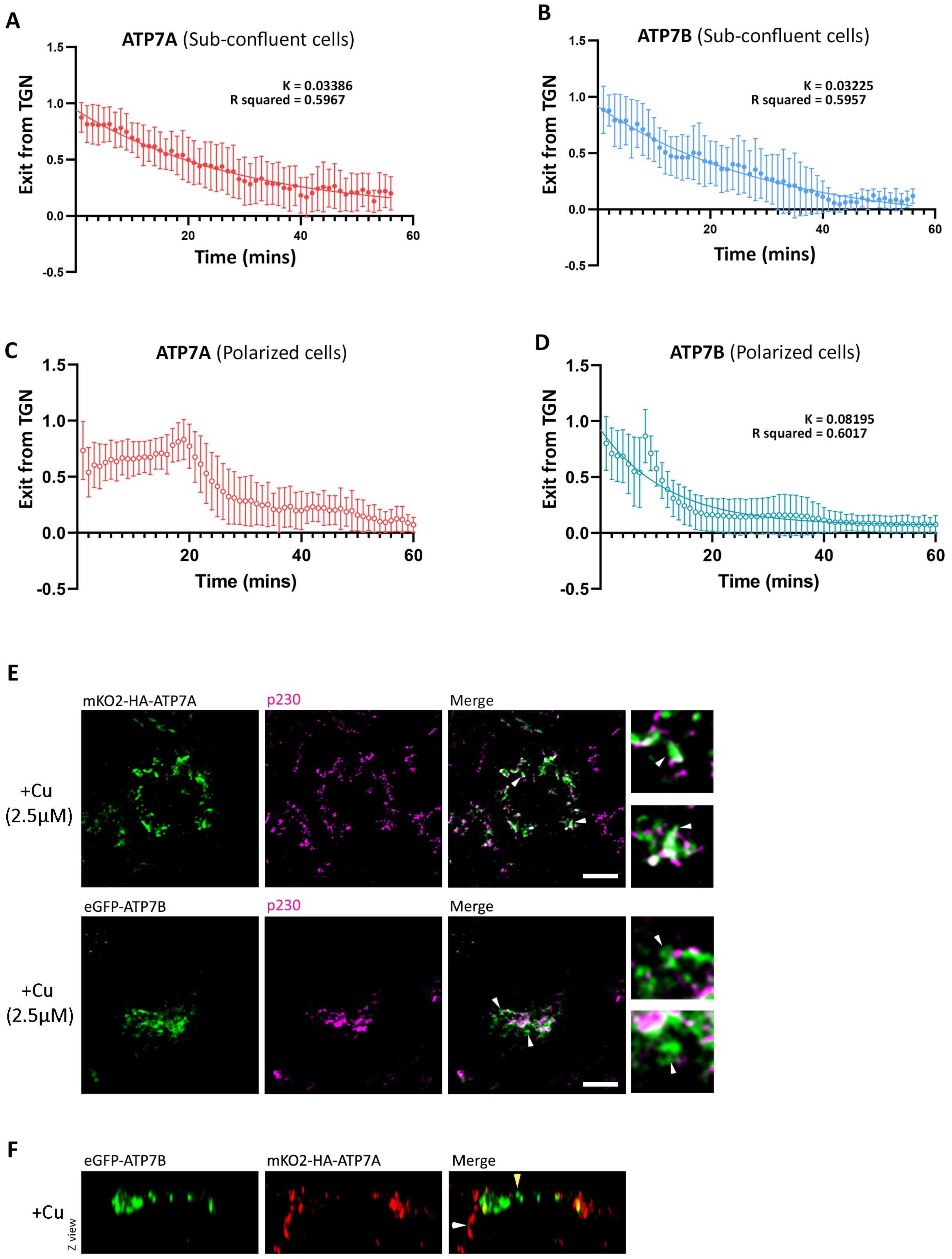

Fig. S1. (A) Fitted curve of decreased pixel count in sub-confluent MDCK cells transfected with mKO2-HA-ATP7A showing its exit rate in response to copper (i.e., k value = 0.03386). (B) Fitted curve of decreased pixel count in sub-confluent MDCK cells transfected with eGFP-ATP7B showing its exit rate in response to copper (i.e., k value = 0.03225). (C) Curve of decreased pixel count in polarized MDCK cells transfected with mKO2-HA-ATP7A showing its exit rate in response to copper. (D) Fitted curve of decreased pixel count in polarized MDCK cells transfected with eGFP-ATP7B showing its exit rate in response to copper (i.e., k value = 0.08195). (E) Representative confocal images of CuCl2 (2.5µM; 30mins) treated cells transfected with either mKO2-HA-ATP7A or eGFP-ATP7B and co-stained with p230. (Arrowheads indicate TGN exited proteins) (F) Confocal images showing co-transfected mKO2-HA-ATP7A and eGFP-ATP7B under copper-treated conditions. Yellow arrow denotes ATP7B localizing at the apical surface. White arrows mark ATP7A at the basolateral surface. Scale bar, 5µm.

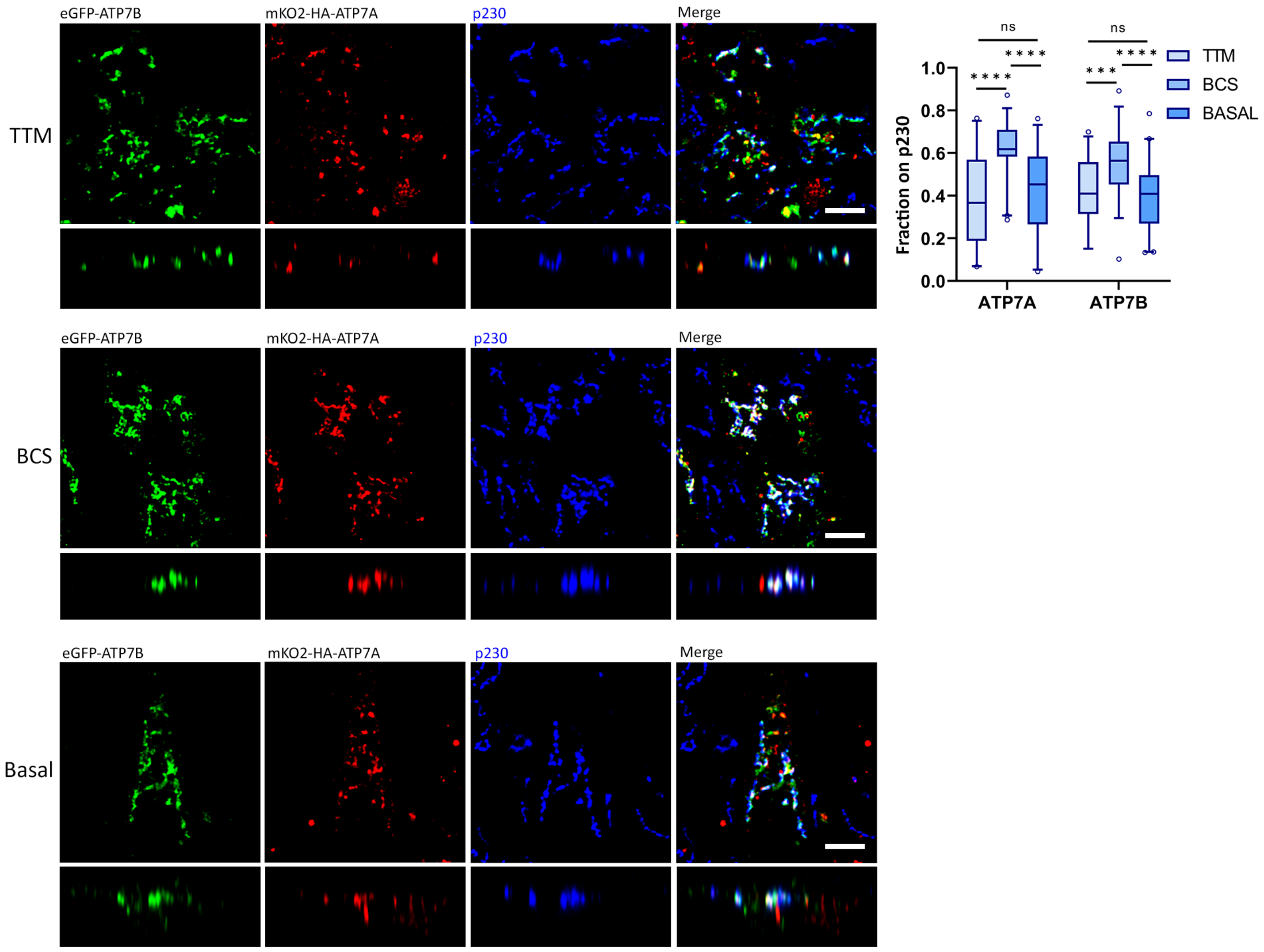

Fig. S2. Polarized MDCK cells showing localization of co-transfected mKO2-HA-ATP7A and eGFP-ATP7B with p230 under limiting copper conditions, i.e., TTM, BCS and Basal. Box-plot showing colocalization quantification (Manders' Colocalization Coefficient) of ATP7A and ATP7B with p230 under different copper conditions, i.e., TTM, BCS and Basal. The box represents the 25th to 75th percentiles, and the median in the middle. The whiskers show the data points within the range of 1.5 × interquartile range (IQR) from the first and third quartile. Sample size (N) for both ATP7A and ATP7B- TTM: 35, BCS: 39, Basal: 43. Scale bar, 5μm.

Journal of Cell Science • Supplementary information

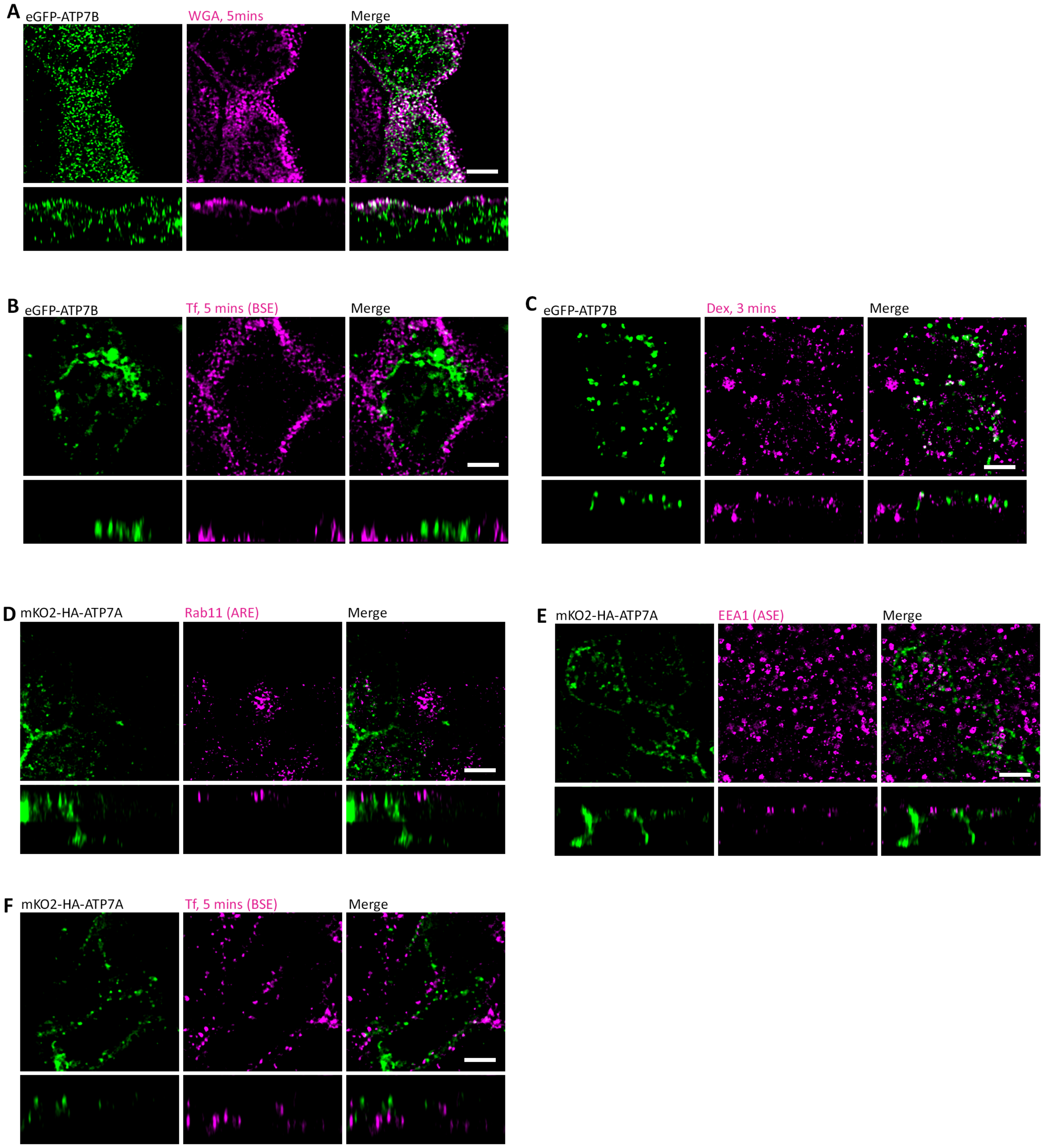

Fig. S3. (A) Confocal image of eGFP-ATP7B (green) co-stained with apically internalized WGA (5 mins) shows presence of ATP7B in ASE in response to copper. (B) Confocal image of eGFP-ATP7B (green) co-stained for BSE (magenta; 5mins Tf Internalization) shows no localization of ATP7B in BSE. (C) Confocal image of eGFP-ATP7B (green) co-stained for early endosomes (magenta; 3mins basolateral dextran uptake) shows no localization of ATP7B in basolateral early endosomes. (D) Confocal image of mKO2-HA-ATP7A (green) co-stained with Rab11 (magenta) shows no localization of ATP7A in ARE (Rab11 positive compartment). (E) Confocal image of mKO-HA-ATP7A co-stained with EEA1 shows no localization of ATP7A in ASE (EEA1 positive compartment). (F) Confocal image of mKO-HA-ATP7A co-stained for BSE shows no localization of ATP7A in Tf (5 min internalized) positive compartments. Scale bar, 5μm.

Journal of Cell Science • Supplementary information

A

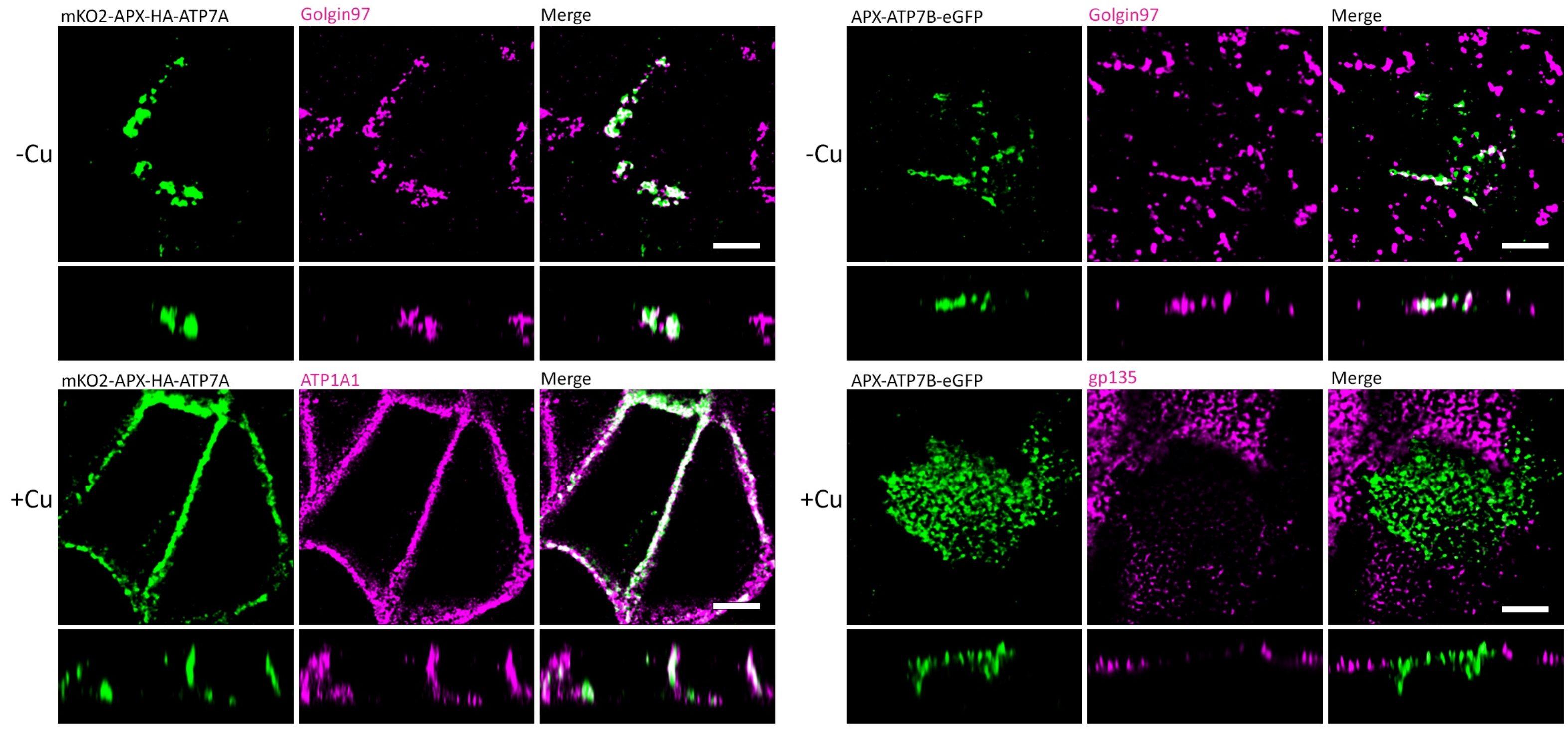

B

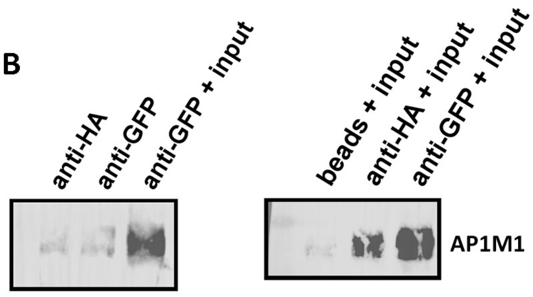

Fig. S4. (A) Polarized MDCK cells showing localization of transfected APEX2-mKO2-HA-ATP7A and APEX2-ATP7B-eGFP where they are localized at TGN in copper deprived conditions and under elevated copper levels traffic to basolateral and apical surfaces respectively, showing wild-type like phenomena. Scale bar, 5µm. (B) Controls for immuno-pulldown of mKO2-HA-ATP7A and eGFP-ATP7B which are immuno-precipitated by anti-HA and anti-GFP antibodies respectively. Isotype and protein control are shown indicating specific pulldowns.

Journal of Cell Science • Supplementary information

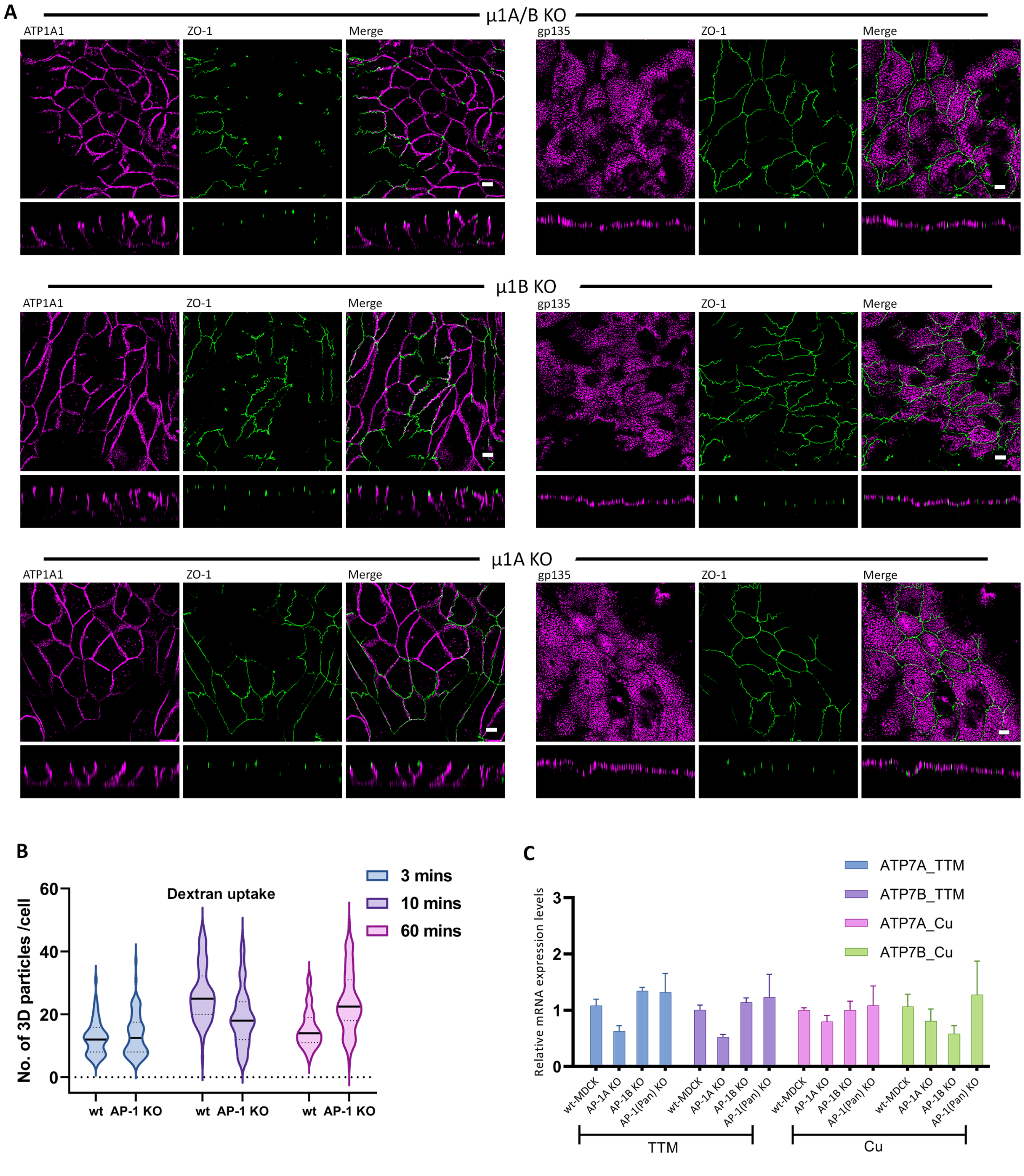

Fig. S5. (A) Images of polarized AP-1 (pan) KO, AP-1B KO, and AP-1A KO MDCK cells marked with tight junctions (stained with ZO-1) and co-stained with respective apical surface (stained with gp135) and basolateral surface (stained with ATP1A1) thus showing their proper polarization. (B) Violin-plots showing no. of 3D-particles of internalized dextran in wild-type and AP-1 (pan) KO polarized MDCK cells. Dextran was internalized for 3 mins, 10 mins and 60 mins showing endocytic endosomal populations. Sample size (N) for 3 mins- wt: 56, AP-1 KO: 93; 10 mins- wt: 79, AP-1 KO: 93; 60 mins- wt: 73, AP-1 KO: 80. (C) Relative mRNA expression levels of ATP7A and ATP7B in wild-type, AP-1A KO, AP-1B KO, and AP-1 (pan) KO MDCK cells under TTM and copper treated conditions. Scale bar, 5µm.

Journal of Cell Science • Supplementary information

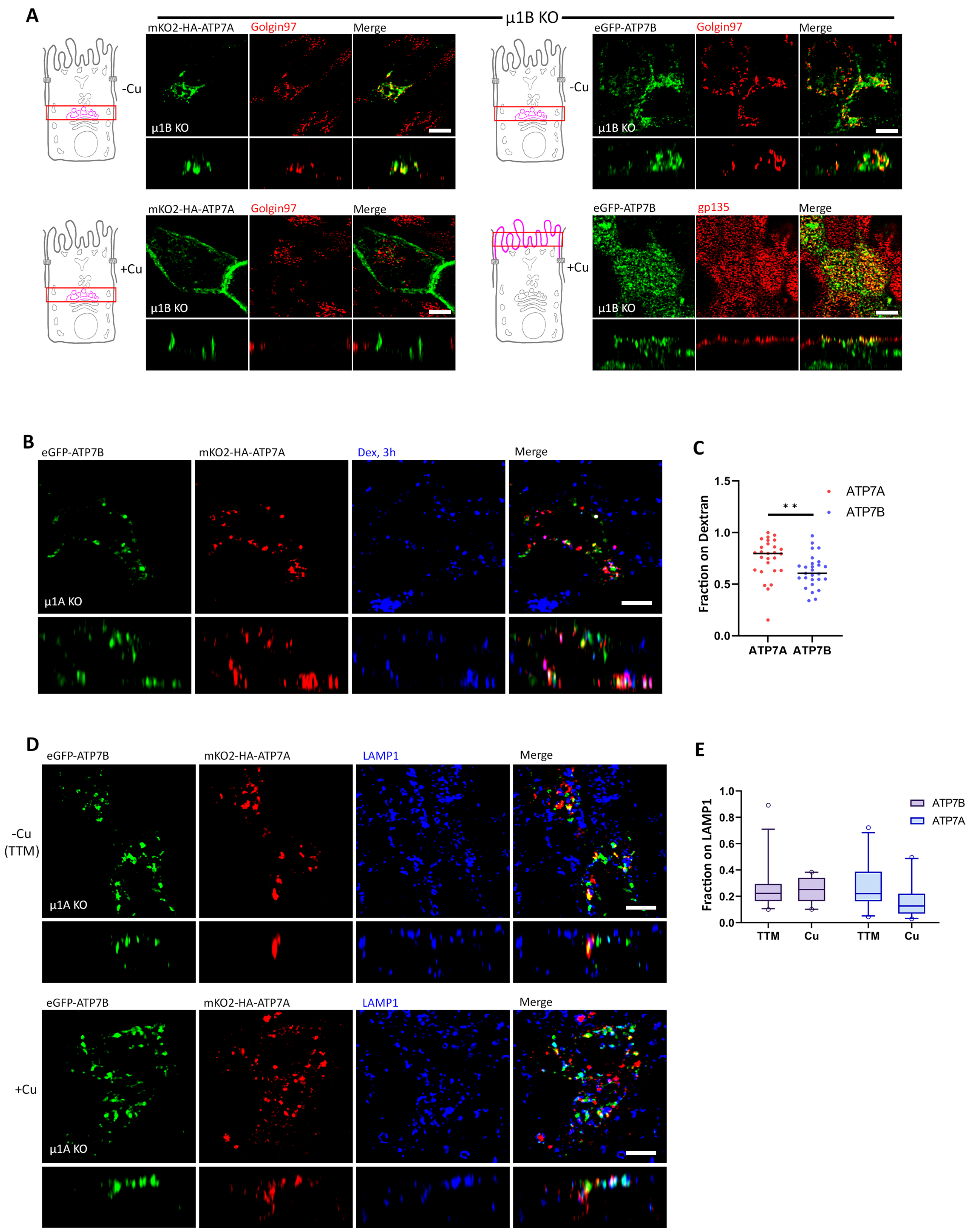

Fig. S6. (A) Confocal image of cells either transfected with mKO2-HA-ATP7A or GFP-ATP7B in AP-1B KO MDCK cells (i.e., µ1B KO cells). Under copper deprived as well as copper-treated conditions both the ATPases exhibit wild-type-like phenomena. (B) Confocal images of polarized AP-1A KO MDCK cells co-transfected with mKO2-HA-ATP7A and eGFP-ATP7B and stained with internalized dextran for 3 hours. (C) Plot showing Manders' Colocalization Coefficient (MCC) of (B), i.e., ATP7A and ATP7B with Dex, 3h. (D) Confocal images of polarized AP-1A KO MDCK cells co-transfected with mKO2-HA-ATP7A and eGFP-ATP7B and stained with LAMP1 in copper deprived and copper treated conditions. (E) Plot showing Manders' Colocalization Coefficient (MCC) of (D) i.e., ATP7A and ATP7B with LAMP1 under TTM and copper treatment. Sample size (N) for both ATP7A and ATP7B at TTM: 28, Cu: 21. Scale bar, 5µm.

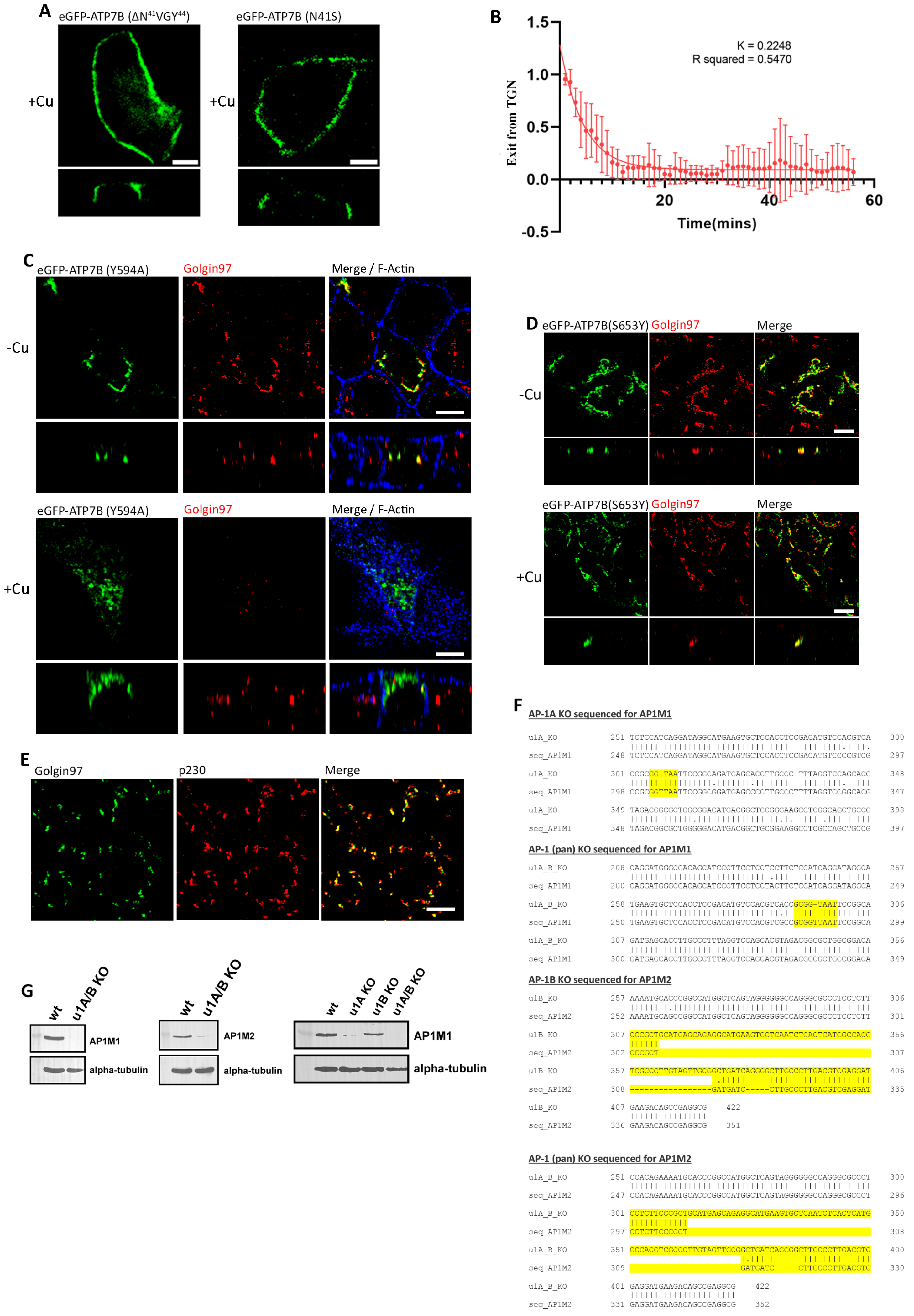
A
eGFP-ATP7B (ΔN41VGY44)
eGFP-ATP7B (N41S)
+Cu
+Cu
B
K = 0.2248
R squared = 0.5470
Exit from TGN
Time(mins)
1.5
1.0
0.5
0.0
-0.5
20
40
60
C
eGFP-ATP7B (Y594A)
Golgin97
Merge / F-Actin
-Cu
+Cu
D
eGFP-ATP7B(S653Y)
Golgin97
Merge
-Cu
+Cu
E
Golgin97
p230
Merge
F
AP-1A KO sequenced for AP1M1
u1A_KO 251 TCTCCATCAGGATAGGCATGAAGTGCTCCACCTCCGACATGTCCACGTCA 300
seq_AP1M1 248 TCTCCATCAGGATAGGCATGAAGTGCTCCACCTCCGACATGTCCCCGTCG 297
u1A_KO 301 CCGCGG-TAATTCCGGCAGATGAGCACCTTGCCC-TTTAGGTCCAGCACG 348
seq_AP1M1 298 CCGCGGTTAATTCCGGCGGATGAGCCCCTTGCCCTTTTAGGTCCGGCACG 347
u1A_KO 349 TAGACGGCGCTGGCGGACATGACGGCTGCGGGAAGCCTCGGCAGCTGCCG 398
seq_AP1M1 348 TAGACGGCGCTGGGGGACATGACGGCTGCGGAAGGCCTCGCCAGCTGCCG 397
AP-1 (pan) KO sequenced for AP1M1
u1A_B_KO 208 CAGGATGGGCGACAGCATCCCTTCCTCCTCCTTCTCCATCAGGATAGGCA 257
seq_AP1M1 200 CAGGATGGGCGACAGCATCCCTTCCTCCTACTTCTCCATCAGGATAGGCA 249
u1A_B_KO 258 TGAAGTGCTCCACCTCCGACATGTCCACGTCACCGCGG-TAATTCCGGCA 306
seq_AP1M1 250 TGAAGTGCTCCACCTCCGACATGTCCACGTCGCCGCGGTTAATTCCGGCA 299
u1A_B_KO 307 GATGAGCACCTTGCCCTTTAGGTCCAGCACGTAGACGGCGCTGGCGGACA 356
seq_AP1M1 300 GATGAGCACCTTGCCCTTTAGGTCCAGCACGTAGACGGCGCTGGCGGACA 349
AP-1B KO sequenced for AP1M2
u1B_KO 257 AAAATGCACCCGGCCATGGCTCAGTAGGGGGGCCAGGGCGCCCTCCTCTT 306
seq_AP1M2 252 AAAATGCAGCCGGCCATGGCTCAGTAGGGGGGCCAGGGCGCCCTCCTCTT 301
u1B_KO 307 CCCGCTGCATGAGCAGAGGCATGAAGTGCTCAATCTCACTCATGGCCACG 356
seq_AP1M2 302 CCCGCT-- 307
u1B_KO 357 TCGCCCTTGTAGTTGCGGCTGATCAGGGGCTTGCCCTTGACGTCGAGGAT 406
seq_AP1M2 308 -----------------GATGATC-----CTTGCCCTTGACGTCGAGGAT 335
u1B_KO 407 GAAGACAGCCGAGGCG 422
seq_AP1M2 336 GAAGACAGCCGAGGCG 351
AP-1 (pan) KO sequenced for AP1M2
u1A_B_KO 251 CCACAGAAAATGCACCCGGCCATGGCTCAGTAGGGGGGCCAGGGCGCCCT 300
seq_AP1M2 247 CCACAGAAAATGCACCCGGCCATGGCTCAGTAGGGGGGCCAGGGCGCCCT 296
u1A_B_KO 301 CCTCTTCCCGCTGCATGAGCAGAGGCATGAAGTGCTCAATCTCACTCATG 350
seq_AP1M2 297 CCTCTTCCCGCT-------------------------------------- 308
u1A_B_KO 351 GCCACGTCGCCCTTGTAGTTGCGGCTGATCAGGGGCTTGCCCTTGACGTC 400
seq_AP1M2 309 -----------------------GATGATC-----CTTGCCCTTGACGTC 330
u1A_B_KO 401 GAGGATGAAGACAGCCGAGGCG 422
seq_AP1M2 331 GAGGATGAAGACAGCCGAGGCG 352
G
wt
u1A/B KO
AP1M1
alpha-tubulin
wt
u1A/B KO
AP1M2
alpha-tubulin
wt
u1A KO
u1B KO
u1A/B KO
AP1M1
alpha-tubulin

Fig. S7. (A) Confocal images of ΔN41VGY44 and N41S of ATP7B mutants shows their basolateral localization in copper-treated conditions. (B) Fitted curve of decreased pixel count of Δ9aa GFP-ATP7B mutant in response to copper showing its dispersion and the exit rate (i.e., k value = 0.2248). (C) Confocal images of Y594A-ATP7B mutant shows wildtype-like copper-mediated trafficking behaviour. (D) Confocal image of S653Y-ATP7B mutant unable to exit TGN in copper-treated condition. (E) Confocal images of polarized MDCK cells stained for Golgin97 and p230 shows colocalization which indicates that they mark the same TGN compartment(s). (F) Sequence alignment of AP1M1 and AP1M2 PCR products from cDNA of AP-1A, AP-1B and AP-1(pan) KO cells show insertions/mismatch/deletions highlighted in yellow, indicating proper knockouts. (G) Immunoblot of AP1M1 and AP1M2 in wild-type and AP-1 knockout MDCK cells. The blots were stripped and re-probed with α tubulin as loading control. Scale bar, 5μm.

Table S1.

Available for download at
https://journals.biologists.com/jcs/article-lookup/doi/10.1242/jcs.261258#supplementary-data

Table S2.

Available for download at
https://journals.biologists.com/jcs/article-lookup/doi/10.1242/jcs.261258#supplementary-data

Table S3.

Available for download at
https://journals.biologists.com/jcs/article-lookup/doi/10.1242/jcs.261258#supplementary-data

Journal of Cell Science • Supplementary information

Table S4. Primers used for ATP7B mutants.

Mutants	Forward Primer	Reverse Primer
ΔF^{37}-E^{45}	5'GGTGGTCTGGATGGCCTG3'	5'ACTCTTCTTCATTGCTGGTTCC3'
$\Delta^{41}NVGY^{44}$	5'GAAGGTGGTCTGGATGGC3'	5'GTCAAAAGCAAAACTCTTCTTCATTG3'
N41S	5'TTTTGCTTTTGACAGTGTTGGCTATGAAG3'	5'CATAGCCAACACTGTCAAAAGCAAAACTC3'
LL1455AA	5'CGCGAATGGCAGGGATGAGGAG	3'GCCGCAGACCACTTGTCCCCATC
Y1376A	5'GCTCAAGTGCGCTAAGAAGCCTGACCTG3'	5'TGCAGGGATGAGAGCACC3'
Y594A	5'TGGCATCACTGCTGCCTCCGTTG3'	5'TTTGTCCTCGTGAGTTTG3'
S653Y	5'TGGAAGAAGTATTTCCTGTGC3'	5'CTGCTTTATTTCCATCTTG3'

Table S5. Primers used for reverse transcription PCR.

Gene	Forward Primer	Reverse Primer
ATP7A	5'CTTGTTGTGAGAGGAATGACGTGTGCC3'	5'CAAGTGGCTTGCTGACCGATCCTTC3'
ATP7B	5'GCTCTTTGTGTTCATTGCCCTGGGG3'	5'CCCGGGGACCACCTTGATGACATC3'
GAPDH	5'CCTGCCGCCTGGAGAAAGC3'	5'TGGAAGAGTGGGTGTCACTGTTG3'

Table S6. Gradient between solution A (5 % acetonitrile-water containing 0.1 % formic acid) and solution B (95 % acetonitrile in water containing 0.1% formic acid) at the flow rate of 300 nl/min.

Time (min)	% sol. B
0	3
41	25
48	40
54	95
60	95

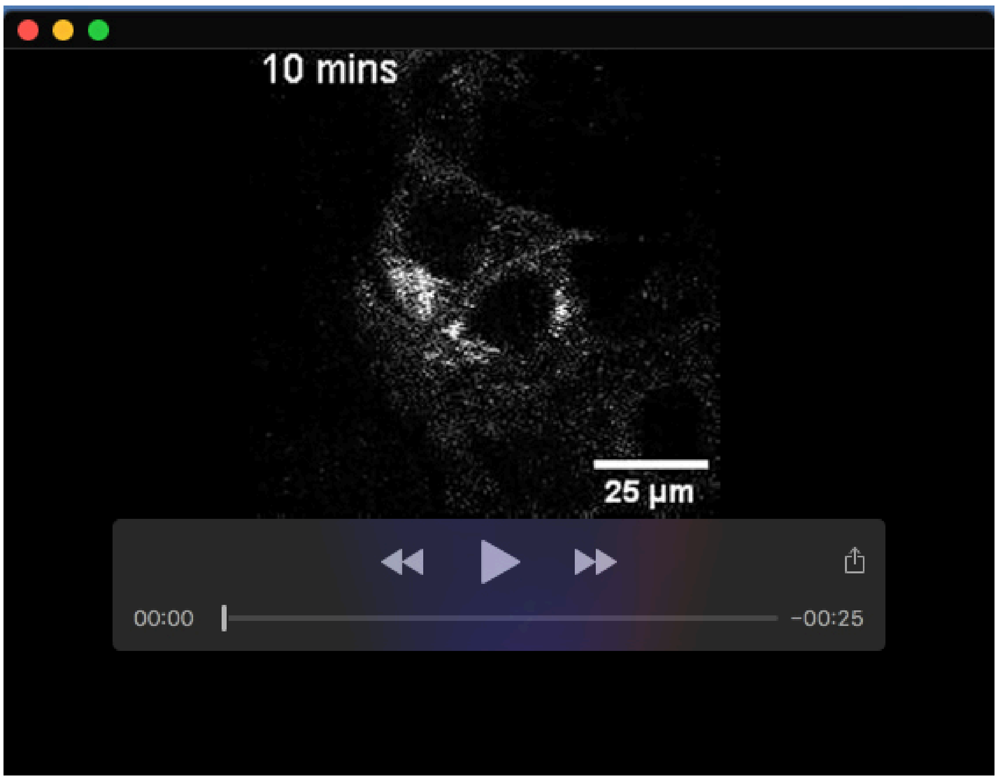

Movie 1. Representative video of dispersion rate of ATP7A from Golgi in response to copper in sub-confluent MDCK cells.

MDCK cells transfected with mKO2-HA-ATP7A were plated on glass bottom confocal dishes. Images of sub-confluent MDCK cells treated with 50µM $CuCl_2$ were captured at an interval of 60 seconds for 1 hour. Pixels having 95% of the signals under basal conditions were considered as Golgi, the disappearance of which represents the Golgi-exit rate.

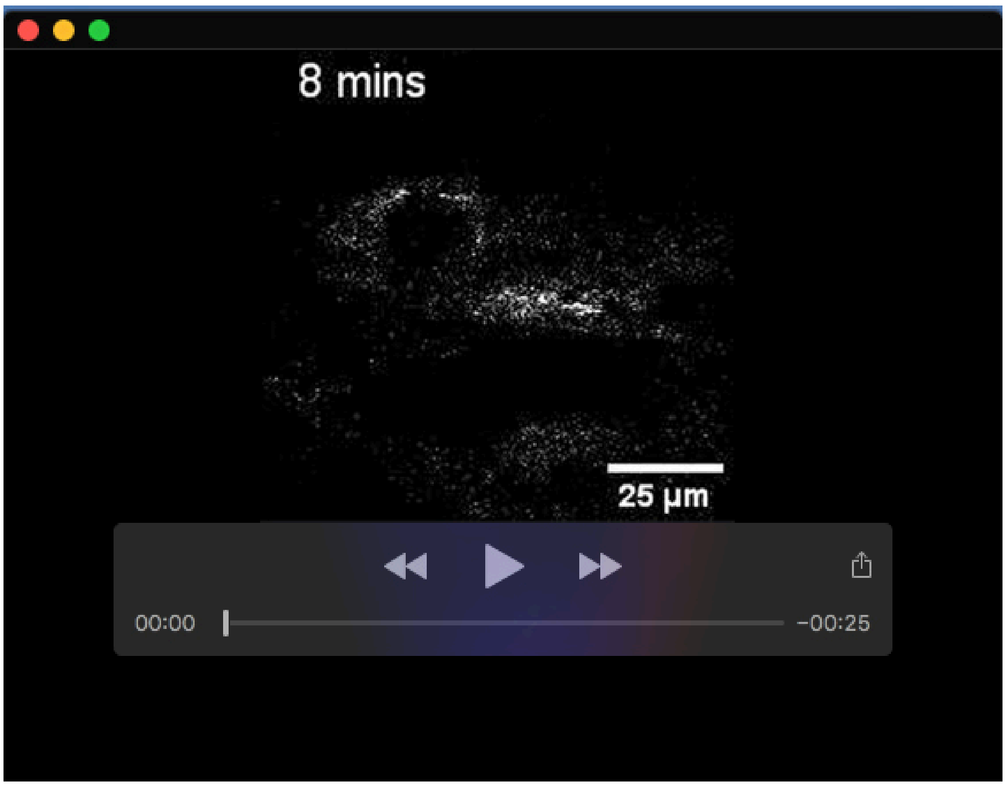

Movie 2. Representative video of dispersion rate of ATP7B from Golgi in response to copper in sub-confluent MDCK cells.

MDCK cells transfected with eGFP-ATP7B were plated on glass bottom confocal dishes. Images of sub-confluent MDCK cells treated with 50µM $CuCl_2$ were captured at an interval of 60 seconds for 1 hour. Pixels having 95% of the signals under basal conditions were considered as Golgi, the disappearance of which represents the Golgi-exit rate.

Journal of Cell Science • Supplementary information

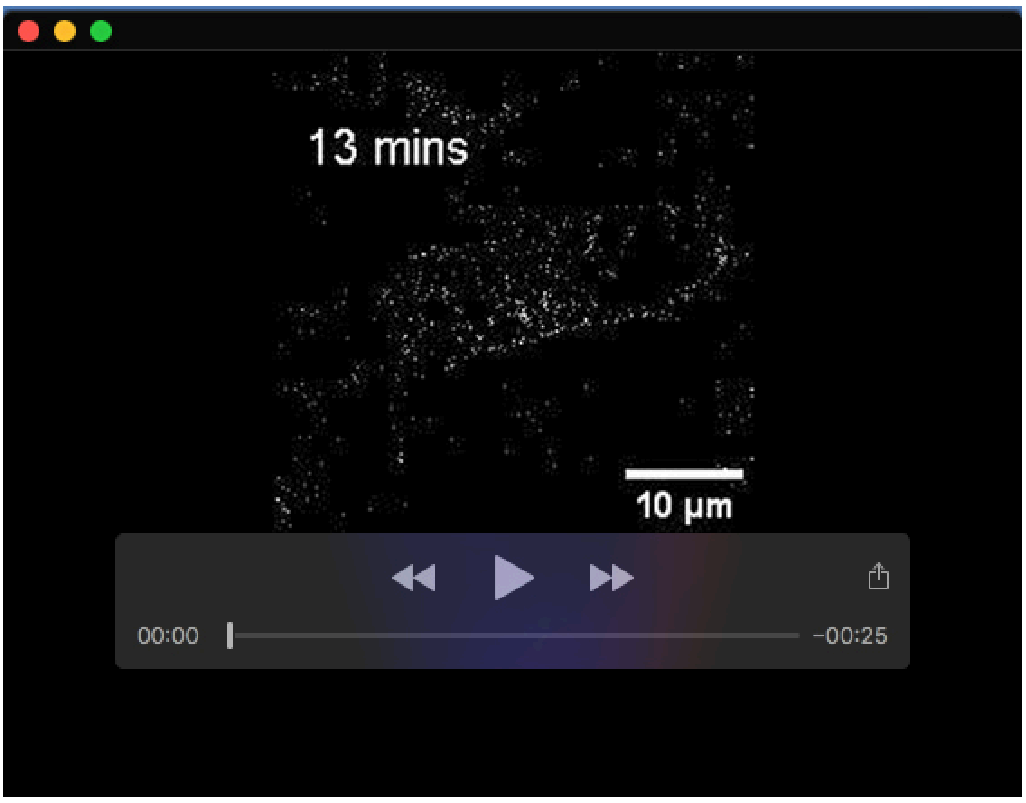

Movie 3. Representative video of dispersion rate of ATP7A from Golgi in response to copper in polarized MDCK cells.

MDCK cells transfected with mKO2-HA-ATP7A were seeded on 0.4µm, 12mm inserts. Images of polarized MDCK cells treated with 50µM $CuCl_2$ were captured at an interval of 60 seconds for 1 hour. Pixels having 95% of the signals under basal conditions were considered as Golgi, the disappearance of which represents the Golgi-exit rate.

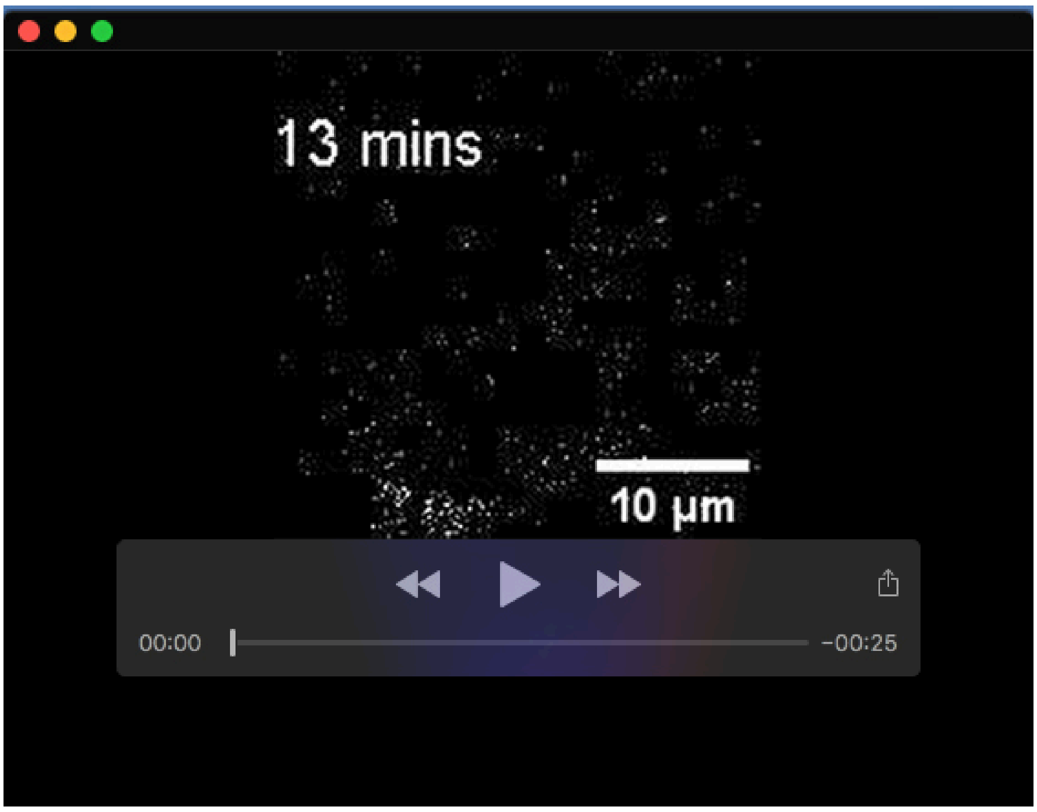

Movie 4. Representative video of dispersion rate of ATP7B from Golgi in response to copper in polarized MDCK cells.

MDCK cells transfected with eGFP-ATP7B were seeded on 0.4µm, 12mm inserts. Images of polarized MDCK cells treated with 50µM $CuCl_2$ were captured at an interval of 60 seconds for 1 hour. Pixels having 95% of the signals under basal conditions were considered as Golgi, the disappearance of which represents the Golgi-exit rate.

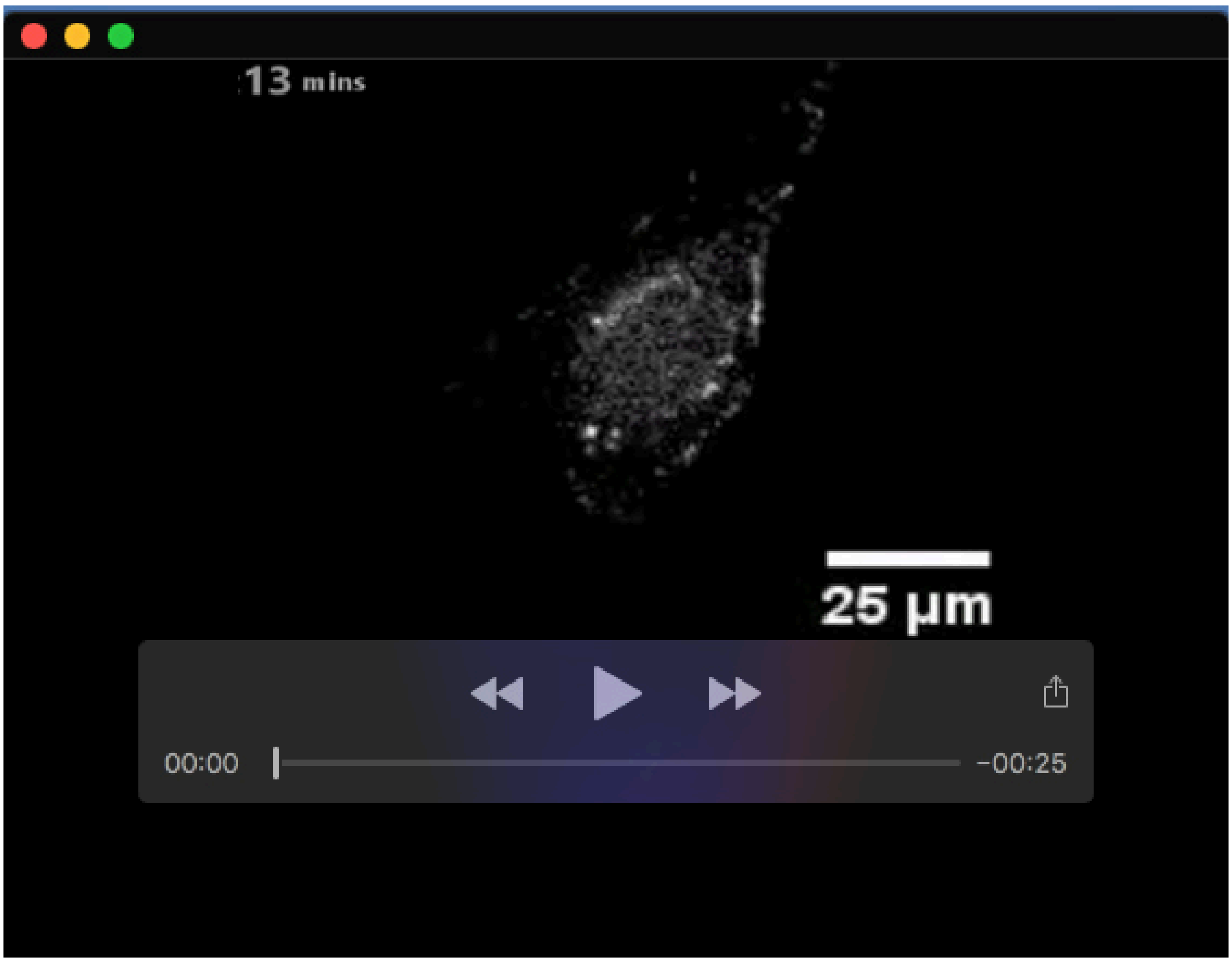

Movie 5. Representative video of dispersion rate of ATP7B ΔF37-E45 mutant from Golgi in response to copper.

MDCK cells transfected with eGFP-ATP7B ΔF^{37}-E^{45} were plated on glass bottom confocal dishes. Images of sub-confluent MDCK cells treated with 50µM $CuCl_2$ were captured at an interval of 60 seconds for 1 hour. Pixels having 95% of the signals under basal conditions were considered as Golgi, disappearance of which represents the Golgi-exit rate.

Journal of Cell Science • Supplementary information
