# Supplementary material for: Regulation of the apico-basolateral trafficking polarity of the homologous copper-ATPases ATP7A and ATP7B
Source: J Cell Sci. 2023 Nov 30;137(5):jcs261258. doi: 10.1242/jcs.261258 (PMC10729821; doi:10.1242/jcs.261258)
Supplement: Supplementary file 1 [file joces-137-261258_review_history.pdf]

Regulation of apico-basolateral trafficking polarity of the homologous copper-ATPases ATP7A and ATP7B

Ruturaj, Monalisa Mishra, Soumyendu Saha, Saptarshi Maji, Enrique Rodriguez-Boulan and Ryan Schreiner and Arnab Gupta
DOI: 10.1242/jcs.261258

Editor: David Bryant

Review timeline

Original submission:	19 April 2023
Editorial decision:	22 May 2023
First revision received:	30 August 2023
Editorial decision:	27 September 2023
Second revision received:	5 October 2023
Accepted:	5 October 2023

Original submission

First decision letter

MS ID#: JOCES/2023/261258

MS TITLE: Regulation of apico-basolateral trafficking polarity of homologous Copper-ATPases ATP7A and ATP7B

AUTHORS: Arnab Gupta, Ruturaj ., Monalisa Mishra, Soumyendu Saha, Saptarshi Maji, Enrique Rodriguez-Boulan, and Ryan Schreiner
ARTICLE TYPE: Research Article

I have now reached a decision on the above manuscript.

The reviewers indicated a general support for your manuscript, but also indicated a number of substantial criticisms that prevent me from accepting the paper at this stage. If you are able to address their criticisms, I would be pleased to see a revised manuscript. We would then return it to the reviewers. To see the reviewers' reports and a copy of this decision letter, please go to: https://submit-jcs.biologists.org and click on the 'Manuscripts with Decisions' queue in the Author Area. (Corresponding author only has access to reviews.)

Of particular importance are elucidation of a) the additional compartments that ATP7A/B localise to in different conditions and whether these compartments are regulated by AP1 (as indicated by reviewers and the literature), b) whether differences in steady state localisation to apical vs basolateral reflects delivery versus retention mechanisms, and c) whether there are clonal differences in the KO approach used, including their polarisation status. It will also be important to indicate how your results align with the literature and existing proposed mechanisms, as indicated by reviewers.

In a resubmission, please provide a point-by-point response detailing how you have dealt with the points raised by the reviewers in the 'Response to Reviewers' box. Please attend to all of the reviewers' comments. If you do not agree with any of their criticisms or suggestions please explain clearly why this is so.Please ensure that you clearly highlight all changes made in the revised manuscript. Please avoid using 'Tracked changes' in Word files as these are lost in PDF conversion.

© 2023. Published by The Company of Biologists under the terms of the Creative Commons Attribution License (https://creativecommons.org/licenses/by/4.0/).

Reviewer 1

Advance summary and potential significance to field

The authors study the intracellular trafficking of two homologous ATPases, ATP7A and ATP7B, which participate in the regulation of the intracellular copper concentration. At low copper, both ATPases accumulate in the TGN. The addition of an excess of copper in the culture medium leads to the accumulation of ATP7A at the basolateral domain and ATP7B at the apical domain of the plasma membrane of polarised epithelial cells. These ATPases are thus a very good model for studying the post-golgi polarised transport.
Ruturaj's manuscript provides new data regarding the trafficking of exogenous versions of ATP7A and ATP7B in polarized MDCK cells. In Fig1, the authors report that in low copper medium both ATP7A and ATP7B are enriched in the TGN. However in response to the addition of copper, ATP7A and ATP7B were found enriched at the basolateral domain or apical domain, respectively. Then the authors show that ATP7A and ATP7B, though both present at the Golgi in low copper medium were segregated within different area of the Golgi. Further, ATP7B was found within multiple endosomal compartments suggesting an indirect route to the apical surface. In contrast, ATP7A was not found in the CRE suggesting a direct route from the Golgi to the basolateral domain. Using the proximity ligation system, the authors identified putative regulators of the intracellular trafficking of the ATPases including AP1. They further investigated the role of AP1 by CRISPR-mediated knock-down of the genes expressing the subunits encoding for mu1A, mu1B or both in MDCK cells. They found that in the KO mu1A cells regardless of the amount of copper, both ATPases were present in vesicular compartments different from each other and from the Golgi. The KO of mu1B had no effect on the steady state localization of both ATPAses in the absence or presence of copper. In the double KO, ATP7A was found intracellularly in the absence of copper but at the plasma membrane in the presence of copper. ATP7B was found at the apical surface with or without copper. Finally, the authors analysed the impact of the double KO on the localization of several ATP7B variants mutated in various sorting motifs.

Comments for the author

The manuscript is well written, the figures are nicely presented, in general the experiments include appropriate controls and the results are clearly described.
It is an interesting piece of work and the results raise many intriguing questions. But this is also due to the fact that there is still a lot missing to draw a solid conclusion.
This work suffers from four major issues that prevent its publication as is: 1) all the analyses are based on a single clone for each KO. The key results for at least one other clone or the puromycin resistant whole population should be shown. 2) the polarized epithelial status of the KO clones is needed. At a minimum the staining for apical, basal, TJ and AJ markers should be shown. Most importantly, 3) all experiments are done at steady state and 4) the vesicular compartments in which the ATPases are found accumulated under different conditions have not been identified. It is therefore impossible to deduce at what level AP1A and AP1B play a role along the ATPases transport pathways. The authors propose leads but there are alternative ways to interpret their results. Thus, there is no substantial mechanistic novelty provided regarding the AP1-regulated post-Golgi transport of the ATPases.
For example, it cannot be concluded whether the enrichment in one compartment is due to retention, as the authors state, or an imbalance of transport in and out of a given compartment to another (between Golgi, vesicular compartments, plasma membrane). Conversely, in AP-1 KO cells (pan), ATP7B may have transited in a non-polarized manner from the Golgi to the apical and basolateral domains to ultimately be accumulated apically by retention. Surface biotinylation experiments (apical or basal) at different times after copper addition would be helpful to determine whether the ATPases pass through the plasma membrane.

Minor
Fig1D : This experiment was performed in sub-confluent non-polarized cells. The experiment should be repeated using a confluent cell monolayer as it was shown that the mechanism of transport out of the Golgi changes with the polarized status of the cells (Jaulin et al.,Dev. Cell, 2007).
Fig4: The control experiments showing that the APEX-2 tagged proteins behave as the WT proteins should be shown as in Fig1C. The presentation of the data in the excel tables could be improved by

© 2023. Published by The Company of Biologists under the terms of the Creative Commons Attribution License (https://creativecommons.org/licenses/by/4.0/).

indicating the pvalues and separating the name of the proteins from the rest of the text from the Fasta headers column.
Fig5 and 6: it should be clearly indicated that the experiments are performed with TTM and it should be noted on the figure panels. The levels of each ATPases in the various conditions should be shown.

Reviewer 2

Advance summary and potential significance to field

The authors studied the trafficking of copper transporters ATP7A and ATP7B in polarized MDCK cells with the aim to elucidate how they remain concentrated at the TGN during basal copper levels and are trafficked to different plasma membrane domains upon copper saturation and describe detailed trafficking itineraries.
They identified clathrin adaptors AP-1A and AP-1B in playing a role.

Comments for the author

In the abstract and throughout the manuscript the authors make a couple of assumptions and conclusion that albeit of interest, are not always fully supported by the presented data. Overall, the images are nice as far as they go. However, the study lacks in clarity and at least in its current presentation seems to be overly convoluted. In addition, often crucial controls are missing and not all conclusions are backed up by solid data.
The latter is already evident in the abstract. For example, the first sentence states 'we suggest a model of apico-basal sorting ….' This is an overstatement as such a generalized model can hardly be deducted from looking at two differentially sorted Cu-ATPases. Further they state '…and identified the machinery involved …'.
There are so many remaining open questions, that it is likely that other parts of the machinery might be identified in the future to name only a few examples.

Major comments and questions:

1) Intracellular localization of the isoforms: unfortunately, figure 2 is missing co-labeling for Golgin97. In the images shown in figure 1, both ATPases co-localize with Golgin97. So where is Golgin97 when they do not co-localize. Could one of the ATPases traffic into lysosomes? What is the evidence that EEA1 should only label ASE? What about co-label with BSE independent of Tf internalization?

2) How does Cu-binding and the associated structural changes influence the availability of the sorting signals? Is it known if sorting signals may become hidden or exposed upon copper binding? What about post-translational modifications on sorting signals? Could they help explain the differences between the studied ATPases?

3) AP1M1 and AP1M2 knock out cells are missing crucial controls: It is not enough just to say that the genomes were sequenced after CRISPR treatment. Were AP1M1 and AP1M2 proteins no longer expressed in the clones? Where there any off-target effects? Could the phenotypes be rescued upon re-expression with respective exogenous copies of AP1M1 and/or AP1M2?

4) Selected APEX2 results that may be of consequence to this study:
a. Unlike ATP7B, ATP7A showed GGA1 as a hit. Is this relevant for TGN retrieval or anterograde movement?
Although there doesn't seem to be a clear consensus as to the specific roles of AP-1A versus GGAs in anterograde and retrograde trafficking [https://doi.org/10.1016/j.cub.2012.07.012], a selective interaction of ATP7A with GGA1 might explain some of the differences between the copper transporters.
b. In addition to AP-1 and AP-2, both transporters also show labeling of AP-3. AP-3 also binds LL-signals and directs trafficking into lysosomes
[https://www.ncbi.nlm.nih.gov/pmc/articles/PMC1170479/pdf/001304.pdf and https://doi.org/10.1083/jcb.200307157]

© 2023. Published by The Company of Biologists under the terms of the Creative Commons Attribution License (https://creativecommons.org/licenses/by/4.0/).

c. Both transporters show labeling of Rab13. Rab13 has been shown to be involved in recycling from endosomes, and in addition to control trafficking between the TGN and CRE [https://doi.org/10.1083/jcb.200802176]. In the latter study, the authors also showed that Rab13 controlled anterograde transport of all cargos that travers recycling endosomes on their way to the surface. It might be relevant for the trafficking itineraries of ATP1A and ATP7B as well.

5) Model and Interpretation of results:
a. Movement from CRE into ARE: The model seems to suggest that there is no traffic between CRE and ARE. This is not in line with data showing a movement from CRE into ARE during pIgA transcytosis [https://doi.org/10.1002/j.1460-2075.1994.tb06513.x], apical-to-basolateral transcytosis of FcRn [https://doi.org/10.1083/jcb.200809122], and the notion that recycling endosomes segregates into functional domains upon polarization [https://doi.org/10.1091/mbc.e05-09-0873]. Please take these observations into considerations for your model.
b. Endosomal changes: Together with AP-2, AP-1A/B are the most abundant clathrin adaptor complexes in cells known for their involvement in trafficking to and fro endosomes. Especially AP-1B has been implied in modifying recycling endosomes [https://doi.org/10.1080/21592799.2015.1074331]. Thus, KO of AP1M1 AP1M2, or both may alter endosomal populations and thus affect Cu-ATPase trafficking independent from active sorting functions.

Specific questions:
1) Figure 1: was copper depleted with TTM or BCS? Based on figure 2, it looks like BCS was used. Please specify.
2) Figure 2: what are the copper concentrations under basal, TTM and BCS treated cells? This should be listed in the figure.
3) Figure 3 and beyond: In the text itself it is said that Tf was internalized for 15 min, but in the methods and the figure legends it is stated that Tn internalization was for 30 min. Which time is correct? This is important as Sheff et al [https://doi.org/10.1083/jcb.145.1.123] showed that Tf receptors accumulates in CRE only after 30 min. The Tf staining in the figures does not look like recycling endosomal staining.

Other comments:
1) The references/discussions to Perez-Bay and Tf receptor trafficking in the absence of AP-1B throughout the result section is not very helpful and rather distracting. Discussion of Tf receptor pathways in relation to the copper ATPases should be restricted to the actual discussion.
2) Please re-think what you present in the main figures and what should be in supplemental material. Often controls or perceived negative results were delegated into supplemental figures when it would be more helpful to have them in the main figures next to the 'positive' resu

Reviewer 3

Advance summary and potential significance to field

This manuscript studies the role of AP1 complexes in the polarized trafficking of the copper transporters ATP7A and ATP7B. Both transporters are known to reside in the TGN under low copper conditions, and are trafficked to the basolateral and apical domains, respectively, upon exposure to increased copper. In this work, fluorescently tagged forms of the transporters were expressed in the cell line MDCK and the routes of trafficking and trafficking machinery were examined.
The data show that these tagged forms reside in the TGN and traffic to the apical and basolateral domains as expected upon exposure to copper and that the exit rates from the TGN are similar with both isoforms. It was found that the transporters partition differently depending upon the copper concentration, but this observation was not pursued. Further experiments examine the impact of different copper concentrations on localization and the subcellular compartments traversed by the transporters. Finally, the impact of pan AP1 and AP1A and AP1B knockout as well as study of known trafficking and disease causing mutants on the polarized trafficking of the transporters is examined. Overall the data are of good quality and provide some new insights into trafficking of these transporters.

© 2023. Published by The Company of Biologists under the terms of the Creative Commons Attribution License (https://creativecommons.org/licenses/by/4.0/).

Comments for the author

While many of the observations are confirmatory of previous studies in non-polarized cells, the subcellular trafficking pathways are of interest. However, there are concerns that could strengthen the manuscript.

1. In Figure 3, it would be helpful if the colocalization with the subcellular markers were performed. While it is stated that ATP7A does not colocalize with the CRE, it appears that there is colocalization in the images provided.
2. As the copper sensitivity provides a "trafficking block" at the TGN, a time course examining the trafficking of the transporters would provide additional detail as to the route taken on the way to the apical and basolateral plasma membrane.
3. It is difficult to follow the logic that AP-1B serves as a backup to AP1A, since knockout of AP1B has the identical phenotype to WT-MDCK and panAP1 knockout still results in trafficking of ATP7B to the apical domain.

An additional concern with the manuscript is the focus on the role of AP1 at the TGN, with no discussion of how this complex could impact trafficking from the endosomal compartment. Since AP1 has known roles in endosomes, and there appears to be an endosomal phenotype in the knockdowns, examining this question is essential.

Minor concern – the references require reformatting.

First revision

<u>Author response to reviewers' comments</u>

<u>Editor:</u>

Of particular importance are elucidation of a) the additional compartments that ATP7A/B localise to in different conditions and whether these compartments are regulated by AP1 (as indicated by reviewers and the literature), b) whether differences in steady state localisation to apical vs basolateral reflects delivery versus retention mechanisms, and c) whether there are clonal differences in the KO approach used, including their polarisation status. It will also be important to indicate how your results align with the literature and existing proposed mechanisms, as indicated by reviewers.

Response: We have addressed all the comments and issues raised by the three reviewers. They are included in the response section below.

<u>Reviewer 1 Advance Summary and Potential Significance to Field:</u>

The authors study the intracellular trafficking of two homologous ATPases, ATP7A and ATP7B, which participate in the regulation of the intracellular copper concentration. At low copper, both ATPases accumulate in the TGN. The addition of an excess of copper in the culture medium leads to the accumulation of ATP7A at the basolateral domain and ATP7B at the apical domain of the plasma membrane of polarised epithelial cells. These ATPases are thus a very good model for studying the post-Golgi polarised transport.

Ruturaj's manuscript provides new data regarding the trafficking of exogenous versions of ATP7A and ATP7B in polarized MDCK cells. In Fig1, the authors report that in low copper medium both ATP7A and ATP7B are enriched in the TGN. However, in response to the addition of copper, ATP7A and ATP7B were found enriched at the basolateral domain or apical domain, respectively.

Then the authors show that ATP7A and ATP7B, though both present at the Golgi in low copper medium, were segregated within different area of the Golgi. Further, ATP7B was found within

© 2023. Published by The Company of Biologists under the terms of the Creative Commons Attribution License (https://creativecommons.org/licenses/by/4.0/).

multiple endosomal compartments suggesting an indirect route to the apical surface. In contrast, ATP7A was not found in the CRE suggesting a direct route from the Golgi to the basolateral domain. Using the proximity ligation system, the authors identified putative regulators of the intracellular trafficking of the ATPases including AP1. They further investigated the role of AP1 by CRISPR-mediated knock-down of the genes expressing the subunits encoding for mu1A, mu1B or both in MDCK cells. They found that in the KO mu1A cells, regardless of the amount of copper, both ATPases were present in vesicular compartments different from each other and from the Golgi. The KO of mu1B had no effect on the steady state localization of both ATPAses in the absence or presence of copper. In the double KO, ATP7A was found intracellularly in the absence of copper but at the plasma membrane in the presence of copper. ATP7B was found at the apical surface with or without copper. Finally, the authors analysed the impact of the double KO on the localization of several ATP7B variants mutated in various sorting motifs.

Reviewer 1 Comments for the Author:

The manuscript is well written, the figures are nicely presented, in general the experiments include appropriate controls and the results are clearly described. It is an interesting piece of work and the results raise many intriguing questions. But this is also due to the fact that there is still a lot missing to draw a solid conclusion.

This work suffers from four major issues that prevent its publication as is:

1) all the analyses are based on a single clone for each KO. The key results for at least one other clone or the puromycin resistant whole population should be shown.

Response: We thank the reviewer for the suggestion. We had stocks of clones that are different for the one used in the experiments. We used the new clones to repeat the essential experiments. AP-1A KO, AP-1B and Pan-AP1 knockouts clones presented identical phenotypes of mKO2-HA- ATP7A and eGFP-ATP7B localizations as the ones we reported in the original manuscript. We have included the data below. We have mentioned this in the revised text (line: 336-338)

[NOTE: We have removed a figure which was provided for the referees in confidence.]

Fig.R1 A, B, C: Colocalization of tagged-ATP7A and ATP7B with Golgin97 in µ1(pan), µ1A and µ1B KO MDCK cells. **R1D** shows localization of ATP7B at the membrane marked by ATP1A1 in µ1(pan) KO cells. Scale bar: 5µm

2) the polarized epithelial status of the KO clones is needed. At a minimum the staining for apical, basal, TJ and AJ markers should be shown.

Response: We have stained the apical (gp135) and the tight junction (ZO1) markers. They exhibit staining that is in concurrence with proper polarization of the MDCK cells. We have included the data as a new fig (Fig. S5A). For basolateral membrane we already had included staining with Na/K-ATPase (ATP1A1) in Fig. S5A, Fig. 6A). (Line: 260-263 and 291-293)

3) Most importantly, all experiments are done at steady state and

Response: We thank the reviewer for pointing it out. We have now repeated the experiments at
(a) <u>4 time points of copper treatment for ATP7B</u> (15 mins, 30 mins, 45 mins and 60 mins). We did not record much differences with Tf internalization (30mins) at these four time points (Fig. 3A, *top panel*). We also found ATP7B in Rab11 and EEA1 positive compartments, suggesting the apical route of ATP7B involves both ARE; a slow recycling compartment (marked by Rab11) and ASE; a fast-recycling compartment (marked by EEA1) respectively (Fig. 3A). With length of copper treatment there was an increase in ATP7B-Rab11 colocalization that reached a steady state at 45 mins. Similarly, ATP7B-EEA1 colocalization peaks at 30 mins and then attained a steady state at 45 and 60 mins of copper treatment (Fig. 3A; *middle and bottom panel*). We also confirmed presence of ATP7B in 5 mins apically internalized WGA compartments (Fig. S3A), which further strengthens the evidence of fast recycling of ATP7B through ASE. (Revised text in line: 191-202)
(b) We also conducted Tf internalization for ATP7A and compared the data with ATP7B. We found that ATP7A exhibits no apparent localization with internalized Tf at 15 and 30 mins. We have

© 2023. Published by The Company of Biologists under the terms of the Creative Commons Attribution License (https://creativecommons.org/licenses/by/4.0/).

included the data in Fig.3B.

4) the vesicular compartments in which the ATPases are found accumulated under different conditions have not been identified. It is therefore impossible to deduce at what level AP1A and AP1B play a role along the ATPases transport pathways. The authors propose leads but there are alternative ways to interpret their results. Thus, there is no substantial mechanistic novelty provided regarding the AP1-regulated post-Golgi transport of the ATPases. For example, it cannot be concluded whether the enrichment in one compartment is due to retention, as the authors state, or an imbalance of transport in and out of a given compartment to another (between Golgi, vesicular compartments, plasma membrane).

Response: We have demonstrated that ATP7B in WT-MDCK cells, exhibit proper colocalization with internalized-Tf, Rab11, EEA1 (Fig.3A). Interestingly, ATP7A does not colocalize with any of these *bona fide* compartment markers in response to copper. This we have already shown in the original manuscript.

However, in AP-1A KO, previously, we conducted a colocalization assay with the Rab-GTPases and internalized Tf. We failed to observe any colocalization between ATP7B/ATP7A and the markers. We thank the reviewers for raising the concern. Hence, we conducted a colocalization assay between ATP7A/ATP7B and internalized Alexa 647-dextran. We noticed that ATP7A exhibits higher localization as compared to ATP7B in dextran-positive compartments (Fig.S6B, S6C).
However, a fraction of ATP7A and ATP7B were still found in uncharacterized compartments. Further, we used LAMP1 and observed that a small fraction of ATP7A and ATP7B localizes on LAMP1 positive endosomes. The new data has now been included as Fig.S6D and Fig.S6E and also in the text (line: 307-315).

Previously AP-1A has been shown to play a key role in regulating apically and basolaterally targeted proteins, whereas AP-1B has been reported for its role in recycling of basolateral proteins (Gravotta et al, Dev Cell. 2012 Apr 17; 22(4): 811–823). Here we show evidence of AP-1A in regulating TGN retention and directionality of Copper ATPases. Previous studies have shown enrichment of proteins in CRE in AP-1A KO cells (Gravotta et al, *Dev Cell*. 2012 Apr 17; 22(4): 811–823). But we did not find such localisation, rather ATP7A and ATP7B are mis-localised to uncharacterized and possibly novel endosomes, some of which positive for dextran.

To address the raised concern of biased enrichment in KO cells, we conducted a dextran uptake assay comparing wt and the Pan-AP-1 knockout MDCK cells at three time points, 3 mins, 10 mins and 1hr (Fig. S5B). We noticed a comparable and unbiased dextran uptake and distribution between the KO and wt cells. This finding has been included in the revised manuscript (line no.: 279-283).

Conversely, in AP-1 KO cells (pan), ATP7B may have transited in a non-polarized manner from the Golgi to the apical and basolateral domains to ultimately be accumulated apically by retention. Surface biotinylation experiments (apical or basal) at different times after copper addition would be helpful to determine whether the ATPases pass through the plasma membrane.

Response: In AP-1 KO (pan) cells, we found ATP7B constitutively accumulated at the plasma membrane in sub-confluent MDCK cells as determined by it colocalization with ATP1A1. We have included the data in figure for reviewer (Fig.R1D).

<u>**Minor**</u>
Fig1D: This experiment was performed in sub-confluent non-polarized cells. The experiment should be repeated using a confluent cell monolayer as it was shown that the mechanism of transport out of the Golgi changes with the polarized status of the cells (Jaulin et al.,Dev. Cell, 2007).

Response: We thank the reviewer for the suggestion. We have conducted the experiment in polarized cells and has now been included in (Fig. 1E, S1C, S1D) and also in the text in the revised manuscript (line:145-149). The videos for TGN exit kinetics of ATP7A and ATP7B in polarized cells have been added as **video 3** and **video 4** respectively.

© 2023. Published by The Company of Biologists under the terms of the Creative Commons Attribution License (https://creativecommons.org/licenses/by/4.0/).

Fig4: The control experiments showing that the APEX-2 tagged proteins behave as the WT proteins should be shown as in Fig1C.

Response: We have confirmed that APEX-2 tag did not affect the localization of the protein and behaved like the endogenous, wt protein. We have tested their localizations in copper chelated as well as elevated copper conditions. The data has been included in (Fig. S4) and also in the text (line: 224-227).

The presentation of the data in the excel tables could be improved by indicating the pvalues and separating the name of the proteins from the rest of the text from the Fasta headers column.

Response: We have conducted a label-free quantification (**LFQ**) of the data and included the p-values as a separate excel dataset (dataset 3). Additionally, we have included a figure showing pathway enrichment (Fig. 4B). The text has been added in the revised manuscript (line: 230-233).

Fig5 and 6: it should be clearly indicated that the experiments are performed with TTM and it should be noted on the figure panels. The levels of each ATPases in the various conditions should be shown.

Response: We have edited as per the suggestions. The level of each ATPases in various conditions are included in the figure. We did not record any recognizable pattern in the levels of expression of the ATPases in the three KO cells at copper chelated or copper treated conditions.

(Fig. S5C). The data has been added to the text in the revised manuscript (line: 283-286).

*****

Reviewer 2 Advance Summary and Potential Significance to Field:

The authors studied the trafficking of copper transporters ATP7A and ATP7B in polarized MDCK cells with the aim to elucidate how they remain concentrated at the TGN during basal copper levels and are trafficked to different plasma membrane domains upon copper saturation and describe detailed trafficking itineraries. They identified clathrin adaptors AP-1A and AP-1B in playing a role.

Reviewer 2 Comments for the Author:

In the abstract and throughout the manuscript the authors make a couple of assumptions and conclusion that albeit of interest, are not always fully supported by the presented data. Overall, the images are nice as far as they go. However, the study lacks in clarity and at least in its current presentation seems to be overly convoluted. In addition, often crucial controls are missing and not all conclusions are backed up by solid data. The latter is already evident in the abstract.

For example, the first sentence states 'we suggest a model of apico-basal sorting ….' This is an overstatement as such a generalized model can hardly be deducted from looking at two differentially sorted Cu-ATPases. Further they state '…and identified the machinery involved …'.

Response: We have edited the statement in the abstract as per the suggestion of the reviewer.

There are so many remaining open questions, that it is likely that other parts of the machinery might be identified in the future to name only a few examples.

Major comments and questions:

1) Intracellular localization of the isoforms: unfortunately, figure 2 is missing co-labeling for Golgin97. In the images shown in figure 1, both ATPases co-localize with Golgin97. So where is Golgin97 when they do not co- localize. Could one of the ATPases traffic into lysosomes? What is the evidence that EEA1 should only label ASE? What about co-label with BSE independent of Tf

© 2023. Published by The Company of Biologists under the terms of the Creative Commons Attribution License (https://creativecommons.org/licenses/by/4.0/).

internalization?

Response: We appreciate the concerns of the reviewer. For co-staining ATP7A, ATP7B and TGN, we used p230 to mark TGN. We conducted colocalization analysis for the three proteins for the three conditions, (a) TTM, (b) BCS and (c) basal. We have included the data in a new figure (Fig. S2) and text (Line: 174-177).

We did not find appreciable localization of the ATPases with LAMP1 (lysosomal marker). The image has been provided below figure for reviewer.

[NOTE: We have removed a figure which was provided for the referees in confidence.]

Fig R2: Colocalization of mKO2-HA-ATP7A and eGFP-ATP7A with lysosomes marked with LAMP1. Scale bar, 5µm.

We agree with the reviewer that EEA1 also labels BSE to a certain extent. However, all the localization studies were imaged from the apical focal planes, less than 2µM from the surface. Also, mentionable since MDCK cells are ~12-14µM in height, it is easy to capture the images at different optical sections avoiding any overlap. Further, we have also conducted as colocalization assay between internalized Alexa-633 WGA (Wheat Germ Agglutinin) and ATP7B. We noticed colocalization between them (Fig. S3A). WGA is only uptaken via the apical membrane for 5mins. Hence, we can safely interpret that ATP7B traffics via ASE. Modified text has been now added in line: 202-203)

To mark BSE (independent of Tf), we also conducted a 3 mins dextran uptake from basolateral side, so that it will mark early sorting endosomes. We did not notice any colocalization between ATP7B and dextran (Fig. S3C). Text has been added in line: 204-207.

We have added all these modifications and additions in the text as well.

2) How does Cu-binding and the associated structural changes influence the availability of the sorting signals? Is it known if sorting signals may become hidden or exposed upon copper binding? What about post- translational modifications on sorting signals? Could they help explain the differences between the studied ATPases?

Response: We thank the reviewer for the valuable comment. Through our own experiments and studies from other groups it has been demonstrated that copper binding to the amino terminal domain and associated structural changes do influence sorting and TGN exit signals. Hasan et al (J Biol Chem ;287(43):36041-50) has shown that regulatory kinase mediated phosphorylation at the Serine stretch (S^{340}-S^{343}) between MBD3 and MBD4 which regulates exit of ATP7B from the TGN in response to copper. Mutating Ser-340/341 in the N-ATP7B individually or together to Ala, Gly, Thr, or Asp shifted the steady-state localization of ATP7B to vesicles, independently of copper levels. Further this region also is responsible for copper-dependent interaction between the amino terminus and the Nucleotide-binding domain.

Braiterman et al (Am J Physiol Gastrointest Liver Physiol. 2009;296(2):G433-44) narrowed down the apical targeting sequence of ATP7B to nine amino acids, F^{37}-E^{45} that constitutes an essential apical targeting determinant for ATP7B in elevated copper and in TGN retention of the protein under low-copper conditions. In this present study by Ruturaj et al, we have utilized multiple Wilson disease causing mutations as well as the Δ F^{37}-E^{45} mutation to characterize possible associated changes at the amino-terminus and post-translational modification that influence TGN exit and subsequent trafficking of ATP7B. Interestingly, ATP7A lacks this stretch or any homologous sequence of these nine amino acids. Mutating these amino acids causes basolateral localization of ATP7B. Based on our experiments (Fig. 7), we hypothesize that ^{41}NVGY^{44} motif that resides in the nine amino acid stretch, F^{37}-E^{45} is the copper-dependent signal sequence for apical targeting of ATP7B, without which the protein behaves similar to ATP7A in terms of targeting and localizes at the basolateral membrane. We have included these discussions in the revised text (line: 461-479).

© 2023. Published by The Company of Biologists under the terms of the Creative Commons Attribution License (https://creativecommons.org/licenses/by/4.0/).

3) AP1M1 and AP1M2 knock out cells are missing crucial controls: It is not enough just to say that the genomes were sequenced after CRISPR treatment. Were AP1M1 and AP1M2 proteins no longer expressed in the clones? Where there any off-target effects? Could the phenotypes be rescued upon re-expression with respective exogenous copies of AP1M1 and/or AP1M2?

Response: Using immunoblot analysis we did not observe expression of AP1M1 in AP-1A KO and AP-1 (pan) KO cells. However, in immunoblot analysis for AP1M2, we could not directly verify in AP-1B KO cells due to cross reactivity with AP1M1. However, in AP-1 (pan) KO cells we did not find expression of AP1M2 (Fig. S7E and line: 709-713). For our previous studies we have tried to rescue the KO phenotypes by exogenously expressing AP1M1 and AP1M2 but overexpression or exogenous expression of these subunits behaves as a dominant negative phenotype (in wildtype cells as well). Hence, we could not perform the rescue experiments.

4) Selected APEX2 results that may be of consequence to this study:

a. Unlike ATP7B, ATP7A showed GGA1 as a hit. Is this relevant for TGN retrieval or anterograde movement? Although there doesn't seem to be a clear consensus as to the specific roles of AP-1A versus GGAs in anterograde and retrograde trafficking [https://doi.org/10.1016/j.cub.2012.07.012], a selective interaction of ATP7A with GGA1 might explain some of the differences between the copper transporters.

Response: We thank the reviewer for the suggestion and agree with the reviewer that GGA1 might be a potential regulator of differential apical-basolateral trafficking. Presently, we are conducting further experiments to explain the differences between ATP7A and ATP7B. However, with current manuscript we focused on differential sorting by AP-1.

b. In addition to AP-1 and AP-2, both transporters also show labeling of AP-3. AP-3 also binds LL-signals and directs trafficking into lysosomes [https://www.ncbi.nlm.nih.gov/pmc/articles/PMC1170479/pdf/001304.pdf and https://doi.org/10.1083/jcb.200307157]

Response: We thank the reviewer for the suggestion. We are further extending our focus on the role of AP-3. AP-3 might play a role in copper independent trafficking of copper ATPases.

c. Both transporters show labeling of Rab13. Rab13 has been shown to be involved in recycling from endosomes, and in addition to control trafficking between the TGN and CRE [https://doi.org/10.1083/jcb.200802176]. In the latter study, the authors also showed that Rab13 controlled anterograde transport of all cargos that travers recycling endosomes on their way to the surface. It might be relevant for the trafficking itineraries of ATP1A and ATP7B as well.

Response: We are also exploring the recycling pathway of copper ATPases in prolonged copper treatment. We believe Rab13 might play a crucial role in recycling of copper ATPases which involves the late anterograde pathway.

5) Model and Interpretation of results:
a. Movement from CRE into ARE: The model seems to suggest that there is no traffic between CRE and ARE. This is not in line with data showing a movement from CRE into ARE during pIgA transcytosis [https://doi.org/10.1002/j.1460-2075.1994.tb06513.x], apical-to-basolateral transcytosis of FcRn [https://doi.org/10.1083/jcb.200809122], and the notion that recycling endosomes segregates into functional domains upon polarization https://doi.org/10.1091/mbc.e05- 09-0873]. Please take these observations into considerations for your model.

Response: We appreciate the concerns raised by the reviewer regarding our proposed model. We have considered the above cited articles and modified our model (Fig. 6G). The previous studies cited by reviewer involves the endocytic and recycling pathway. But our model is for the bio-synthetic pathway of ATP7A and ATP7B that originates at the TGN and undergoes copper mediated anterograde trafficking towards the plasma membranes. We did not observe any movement of ATP7B into ARE in AP-1B KO cells unlike previous studies, where recycling proteins like TfR mislocalize into apical route in AP-1B KO cells. In addition to that, we found the segregation of

© 2023. Published by The Company of Biologists under the terms of the Creative Commons Attribution License (https://creativecommons.org/licenses/by/4.0/).

apically targeted ATP7B and basolateral targeted ATP7A happens at the TGN. Under basal condition both the copper ATPases are distinctly located. Further co-staining with TGN marker we observed a non-overlapping distinct ATP7A and ATP7B with p230 (TGN marker). (Revised Fig. S2).

b. *Endosomal changes: Together with AP-2, AP-1A/B are the most abundant clathrin adaptor complexes in cells known for their involvement in trafficking to and fro endosomes. Especially AP-1B has been implied in modifying recycling endosomes https://doi.org/10.1080/21592799.2015.1074331]. Thus, KO of AP1M1, AP1M2, or both may alter endosomal populations and thus affect Cu-ATPase trafficking independent from active sorting functions.*

Response: We thank the reviewer for the point raised regarding changes in endosomal population. We have conducted dextran uptake assay comparing wt and the Pan AP-1 knockout MDCK cells at three time points, 3 mins, 10 mins and 1hr and analysed the number of dextran positive compartment. We noticed a comparable and unbiased (random) dextran uptake and distribution between the KO and wt cells. We have included this data in the new figure (Fig. S5B). We have added the text in line no. 279-283.

We can safely deduce that AP-1 actively sorts the ATPases into distinct trafficking routes. Further this is supported by the Co-IP experiment where we observe interaction of AP1M1 with ATP7A as well as ATP7B (Fig. 4D).

Specific questions:

1) Figure 1: was copper depleted with TTM or BCS? Based on figure 2, it looks like BCS was used. Please specify.

Response: We used BCS to deplete copper. We have now mentioned it in the modified figure (Fig.1).

2) Figure 2: what are the copper concentrations under basal, TTM and BCS treated cells? This should be listed in the figure.

Response: We have mentioned the copper concentrations in Fig.2D. The text is accordingly is mentioned in line no. 177-180.

3) Figure 3 and beyond: In the text itself it is said that Tf was internalized for 15 min, but in the methods and the figure legends it is stated that Tn internalization was for 30 min. Which time is correct? This is important as Sheff et al [https://doi.org/10.1083/jcb.145.1.123] showed that Tf receptors accumulates in CRE only after 30 min. The Tf staining in the figures does not look like recycling endosomal staining.

Response: We thank the reviewer for pointing this out. To mark CRE all the experiments are done at 30 mins Tf uptake and to mark only BSE 5 mins Tf uptake was performed. The text has been modified accordingly. Line: 213-215

Other comments:

1) *The references/discussions to Perez-Bay and Tf receptor trafficking in the absence of AP-1B throughout the result section is not very helpful and rather distracting. Discussion of Tf receptor pathways in relation to the copper ATPases should be restricted to the actual discussion.*

Response: We understand the concerns of the reviewer. However, TfR being a recycling protein mislocalizes to ARE in absence of AP-1B. But ATP7B in late secretory pathway does not follow that path; rather there is no dispersion of ATPB from CRE further to the ARE in AP-1B KO. We have now elucidated our statement in the text (line: 325-328)

2) *Please re-think what you present in the main figures and what should be in supplemental material. Often, controls or perceived negative results were delegated into supplemental figures when it would be more helpful to have them in the main figures next to the 'positive' result.*

Response: We agree that controls are essential and have reorganized the figures accordingly.

© 2023. Published by The Company of Biologists under the terms of the Creative Commons Attribution License (https://creativecommons.org/licenses/by/4.0/).

However, due to space constrains, we could not move all the controls to main figures. However, we have now showed all the controls elaborately in supplementary and main figures.

*****

Reviewer 3 Advance Summary and Potential Significance to Field:

This manuscript studies the role of AP1 complexes in the polarized trafficking of the copper transporters ATP7A and ATP7B. Both transporters are known to reside in the TGN under low copper conditions, and are trafficked to the basolateral and apical domains, respectively, upon exposure to increased copper. In this work, fluorescently tagged forms of the transporters were expressed in the cell line MDCK and the routes of trafficking and trafficking machinery were examined.
The data show that these tagged forms reside in the TGN and traffic to the apical and basolateral domains as expected upon exposure to copper and that the exit rates from the TGN are similar with both isoforms. It was found that the transporters partition differently depending upon the copper concentration, but this observation was not pursued. Further experiments examine the impact of different copper concentrations on localization and the subcellular compartments traversed by the transporters. Finally, the impact of pan AP1 and AP1A and AP1B knockout as well as study of known trafficking and disease-causing mutants on the polarized trafficking of the transporters is examined.
Overall the data are of good quality and provide some new insights into trafficking of these transporters.

Reviewer 3 Comments for the Author:
While many of the observations are confirmatory of previous studies in non-polarized cells, the subcellular trafficking pathways are of interest. However, there are concerns that could strengthen the manuscript.
1. In Figure 3, it would be helpful if the colocalization with the subcellular markers were performed. While it is stated that ATP7A does not colocalize with the CRE, it appears that there is colocalization in the images provided.

Response: We thank the reviewer for the suggestions. We have now included the colocalization studies and subsequent analysis for subcellular markers in (Fig. 3A) and the revised text (line: 191-205).
For ATP7A, we conducted colocalization experiments with internalized Tf that marks CRE and BSE. Remarkably, ATP7A and 30 mins internalized Tf are juxtaposed, we did not find appreciable colocalization with CRE as compared to ATP7B. This has been included in (Fig. 3B) and revised text (line no. 206-208).

2. As the copper sensitivity provides a "trafficking block" at the TGN, a time course examining the trafficking of the transporters would provide additional detail as to the route taken on the way to the apical and basolateral plasma membrane.

Response: For ATP7B, we have conducted the time course experiments for four timepoints (15 mins, 30 mins, 45 mins, 60 mins). We did not observe much change with Tf internalization at these four time points. We also found ATP7B in Rab11 and EEA1 positive compartments, suggesting the apical route of ATP7B involves both Apical Recycling Endosomes (ARE); a slow recycling compartment (marked by Rab11) and Apical Sorting Endosomes (ASE); a fast-recycling compartment (marked by EEA1) respectively (revised Fig. 3A). With length of copper treatment there was an increase in ATP7B-Rab11 colocalization that reached a steady state at 45 mins.
Similarly, ATP7B-EEA1 colocalization peaks at 30 mins and then attained a steady state at 45 and 60 mins of copper treatment (Fig. 3A; *middle and bottom panel*). The revised text has been included in lines 191-202.

3. It is difficult to follow the logic that AP-1B serves as a backup to AP1A, since knockout of AP1B has the identical phenotype to WT-MDCK and panAP1 knockout still results in trafficking of ATP7B to the apical domain.

© 2023. Published by The Company of Biologists under the terms of the Creative Commons Attribution License (https://creativecommons.org/licenses/by/4.0/).

Response: We agree with the reviewer, AP-1B doesn't serve as a backup, rather it is works as a check point for sorting in absence of AP-1A. We have mentioned this part of our proposed model in line 442-445.

An additional concern with the manuscript is the focus on the role of AP1 at the TGN, with no discussion of how this complex could impact trafficking from the endosomal compartment. Since AP1 has known roles in endosomes, and there appears to be an endosomal phenotype in the knockdowns, examining this question is essential.

Response: We completely agree with the reviewer that AP-1 can regulate retention at and trafficking from the endosomal compartments. As a crucial example of such phenomenon, we have cited and discussed the findings from Raposo's group where Delevoye et al demonstrated that AP-1, and its interacting motor, KIF13A, cooperate to generate recycling endosomal domains that are specified for communication with melanosomes. AP-1 and the KIF cooperate in cargo sorting and in positioning endosomes at the cell periphery near melanosomes, permitting the formation of interorganellar tubular connections (Delevoye et al, *J Cell Biol*. 2009 Oct 19; 187(2): 247–264). We have discussed it in line: 455-460.

Minor concern – the references require reformatting.

Response: We have now reformatted the existing as well as the new references appropriately.

Second decision letter

MS ID#: JOCES/2023/261258

MS TITLE: Regulation of apico-basolateral trafficking polarity of homologous Copper-ATPases ATP7A and ATP7B

AUTHORS: Arnab Gupta, Ruturaj ., Monalisa Mishra, Soumyendu Saha, Saptarshi Maji, Enrique Rodriguez-Boulan, and Ryan Schreiner
ARTICLE TYPE: Research Article

We have now reached a decision on the above manuscript.

To see the reviewers' reports and a copy of this decision letter, please go to: https://submit-jcs.biologists.organd click on the 'Manuscripts with Decisions' queue in the Author Area. (Corresponding author only has access to reviews.)

Although there is a general consensus of an improvement in your manuscript, the reviewers raise a number of criticisms that prevent me from accepting the paper at this stage. I am sympathetic to having an additional round of manuscript revision. To this end, I believe that a number of the reviewer comments can be addressed by text clarification, which may include toning down some of your conclusions. However, some of these will require additional experimental work, which I hope you have to hand, or can perform without arduous additional work. My interpretation is that the staining the reviewers indicate are not arduous. If some experiments are indeed arduous, I would at very minimum suggest discussion of additional interpretations or caveats in your data, as a mechanism to address remaining reviewer concerns. I do expect data to be presented for validation of KO, which as the reviewer points out the manuscript indicates you have performed. I also note that Reviewer 3 indicates that they feel the movies are superfluous. As neither other reviewer shared this opinion, I suggest that these remain.

If you can address these concerns and you think that you can deal satisfactorily with the criticisms on revision, I would be pleased to see a revised manuscript.

Please ensure that you clearly highlight all changes made in the revised manuscript. Please avoid using 'Tracked changes' in Word files as these are lost in PDF conversion.

© 2023. Published by The Company of Biologists under the terms of the Creative Commons Attribution License (https://creativecommons.org/licenses/by/4.0/).

I should be grateful if you would also provide a point-by-point response detailing how you have dealt with the points raised by the reviewers in the 'Response to Reviewers' box. Please attend to all of the reviewers' comments. If you do not agree with any of their criticisms or suggestions please explain clearly why this is so.

Reviewer 1

Advance summary and potential significance to field

The authors have responded to my comments by providing new controls and results that reinforce the initial observations. Though failing to provide a general mechanistic model describing the roles of ATP1B and ATP1A, the manuscript still provides solid new data, using the copper transporters ATP7A and ATP7B as appropriate study models, that nicely feeds our reflexion in the field of polarized trafficking. For this reason, I consider that the manuscript is now publishable. I would, however, recommend including the figures for reviewers in the supplementary data of the final version.

Comments for the author

The authors have responded to my comments by providing new controls and results that reinforce the initial observations. Though failing to provide a general mechanistic model describing the roles of ATP1B and ATP1A, the manuscript still provides solid new data, using the copper transporters ATP7A and ATP7B as appropriate study models, that nicely feeds our reflexion in the field of polarized trafficking. For this reason, I consider that the manuscript is now publishable. I would, however, recommend including the figures for reviewers in the supplementary data of the final version.

Reviewer 2

Advance summary and potential significance to field

This is a revised manuscript that albeit improved over the previous submission, leaves much room for improvement and some of my concerns remain.

Comments for the author

Areas that need further improvement:

1) I am still not satisfied with the characterization of the KO lines. I understand the problems with AP1M1 or AP1M2-specific antibodies. However, the authors say they confirmed KO by sequencing. Could those results be shown? It should be possible to run PCRs with either primers that are specific for the alterations or primers that give differently sized PCR fragments. If none of these methods work, then there remains the option to show lack of function through physiological tests as for example sorting of transferrin receptors and the likes.
2) Staining against TGN markers Golgin-97 and p230/Golgin-245 are used interchangeably. However, since the authors would like to conclude that ATP7A and APT7B reside in different TGN domains, it would be necessary to confirm that the reference TGN markers are indeed in the same TGN domain. This control is missing.
3) In Figure 3: The co-localizations of ATP7B with Tf (30 min), Rab11, and EEA1 are in highly saturated areas. Judging from the labeling patterns, the only co-localization that seems real is the one with Rab11. To further explore if APT7B travels through endosomal populations, please see Cresawn et al.: 10.1038/sj.emboj.7601813 for suggestions.
4) Regarding Figure 3: Was ATP7A labeling tested against 5 min Tf uptake? EEA1 in the images seems to only stain apical early endosomes with a lack of EEA1-positive structures underlying the basolateral membrane. Thus, there needs to be further proof to conclude that ATP7A does not move through early endosomes.

© 2023. Published by The Company of Biologists under the terms of the Creative Commons Attribution License (https://creativecommons.org/licenses/by/4.0/).

5) In Figure 4D: Where is the negative control? Also, since an AP1M1 antibody was used, this shows co-precipitation with AP-1A only at best and not pan-AP-1 as stated in the text.

Reviewer 3

Advance summary and potential significance to field

This is a revised submission of a manuscript that describes polarized sorting of the copper ATPases ATP7A and ATP7B. The authors find that the AP1 complex is involved in the polarized distribution of these molecules with different roles for copper in directing the trafficking. They further identify motifs in the amino terminal domain of ATP7B that are mutated in human genetic disease.

Comments for the author

The authors have somewhat responded to the previous critique. I do have some questions about how the results are interpreted.
1. It is stated the ATP7A does not traverse an endosomal compartment. However, the colocalization of ATP7A with internalized transferrin is nearly the same as that seen with ATP7B and EEA1, which is stated as evidence for ATP7B traversing the ASE.
2. Since AP1B knockout results in normal targeting of both transporters, how can it be that, as stated in the abstract "AP-1B governs polarized trafficking of ATP7B solely."
3. While an endosomal trafficking route is discussed in the discussion, the manuscript focuses on the role of AP1 in retention of the these transporters in the TGN. I do not think that the data can distinguish between a role for AP1 in TGN retention and a failure to retrieve the transporters from the plasma membrane or endosomal compartments.
4. It is my opinion that the videos are superfluous.

Second revision

Author response to reviewers' comments

Editor's comments

Although there is a general consensus of an improvement in your manuscript, the reviewers raise a number of criticisms that prevent me from accepting the paper at this stage. I am sympathetic to having an additional round of manuscript revision. To this end, I believe that a number of the reviewer comments can be addressed by text clarification, which may include toning down some of your conclusions. However, some of these will require additional experimental work, which I hope you have to hand, or can perform without arduous additional work. My interpretation is that the staining the reviewers indicate are not arduous. If some experiments are indeed arduous, I would at very minimum suggest discussion of additional interpretations or caveats in your data, as a mechanism to address remaining reviewer concerns. I do expect data to be presented for validation of KO, which as the reviewer points out the manuscript indicates you have performed. I also note that Reviewer 3 indicates that they feel the movies are superfluous. As neither other reviewer shared this opinion, I suggest that these remain.

Response: We have carried out the experiments that were suggested by the reviewers. In the revised version, we have also added the sequence alignments that confirms AP knockouts in all the cell lines. All the changes have been highlighted in yellow in the text. A point-by-point response follows:

Response to reviewers:

Reviewer 1 Advance Summary and Potential Significance to Field:
The authors have responded to my comments by providing new controls and results that reinforce the initial observations. Though failing to provide a general mechanistic model describing the roles

© 2023. Published by The Company of Biologists under the terms of the Creative Commons Attribution License (https://creativecommons.org/licenses/by/4.0/).

of ATP1B and ATP1A, the manuscript still provides solid new data, using the copper transporters ATP7A and ATP7B as appropriate study models, that nicely feeds our reflexion in the field of polarized trafficking. For this reason, I consider that the manuscript is now publishable. I would, however, recommend including the figures for reviewers in the supplementary data of the final version.

Reviewer 1 Comments for the Author:

The authors have responded to my comments by providing new controls and results that reinforce the initial observations. Though failing to provide a general mechanistic model describing the roles of ATP1B and ATP1A, the manuscript still provides solid new data, using the copper transporters ATP7A and ATP7B as appropriate study models, that nicely feeds our reflexion in the field of polarized trafficking. For this reason, I consider that the manuscript is now publishable. I would, however, recommend including the figures for reviewers in the supplementary data of the final version.

Response: We agree that having the figures for reviewers in the manuscript supplementary data will help. However, given the space constrains and the number of figures allowed, we could not include them. We have mentioned at the journal site that all the data (raw and curated) will be available upon request. We thank the reviewer for the valuable comments.

*****

Reviewer 2 Advance Summary and Potential Significance to Field: This is a revised manuscript that albeit improved over the previous submission, leaves much room for improvement and some of my concerns remain.

Reviewer 2 Comments for the Author:

Areas that need further improvement:

1) I am still not satisfied with the characterization of the KO lines. I understand the problems with AP1M1 or AP1M2-specific antibodies. However, the authors say they confirmed KO by sequencing. Could those results be shown? It should be possible to run PCRs with either primers that are specific for the alterations or primers that give differently sized PCR fragments. If none of these methods work, then there remains the option to show lack of function through physiological tests as for example sorting of transferrin receptors and the likes.

Response: We appreciate the concerns by the reviewer. We have attached the sequencing alignment data for the knockouts, where insertions/deletions/mismatch are clearly visible (highlighted) indicating proper knockout of the desired protein i.e., μ1A and μ1B (Fig. S7F). We have modified the text (line. 739-744).

2) Staining against TGN markers Golgin-97 and p230/Golgin-245 are used interchangeably. However, since the authors would like to conclude that ATP7A and APT7B reside in different TGN domains, it would be necessary to confirm that the reference TGN markers are indeed in the same TGN domain. This control is Missing.

Response: We thank the reviewer for raising this issue. Previously we failed to mention that in double transfected cells, we have observed weak staining of Golgin97. For that purpose, we have used p230 instead of Golgin97. We have now performed the experiment where we observe p230 and Golgin-97 to colocalize. The new figure has been incorporated as Fig.S7E. Additionally, these two studies (Cui et al, *Cell Biol Int 2019*, https://doi.org/10.1002/cbin.11118, Lu et al, *Traffic 2006*, https://doi.org/10.1111/j.1600-0854.2006.00473.x) reported that they stain the same compartment of TGN. We have added this in the text (line. 536-538).

3) In Figure 3: The co-localizations of ATP7B with Tf (30 min), Rab11, and EEA1 are in highly saturated areas. Judging from the labeling patterns, the only co-localization that seems real is the one with Rab11. To further explore if APT7B travels through endosomal populations, please see

© 2023. Published by The Company of Biologists under the terms of the Creative Commons Attribution License (https://creativecommons.org/licenses/by/4.0/).

Cresawn et al.:10.1038/sj.emboj.7601813 for suggestions.

Response: We appreciate the comment by the reviewer. But we want to clarify that all the images were adjusted with "Over-/Underexposure (Leica LasX software)" settings keeping both saturated and lowest grey value areas. And the provided images are adjusted uniformly for better representation purposes. All the colocalization analysis were performed from raw images.
We also thank the reviewer for the valuable suggestion regarding the future prospects of the study.

A representative raw image on which analyses were performed is inserted below:

[NOTE: We have removed a figure which was provided for the referees in confidence.]

4) Regarding Figure 3: Was ATP7A labeling tested against 5 min Tf uptake? EEA1 in the images seems to only stain apical early endosomes with a lack of EEA1-positive structures underlying the basolateral membrane. Thus, there needs to be further proof to conclude that ATP7A does not move through early Endosomes.

Response: We thank the reviewer for the concern. As 30 min Tf uptake also marks the 5 min internalised compartments, we did not include the image with 5 min uptake. But due to concerns raised by the reviewer, we have included the same. Fig.S3F and in the text (Line. 226-228)

5) In Figure 4D: Where is the negative control? Also, since an AP1M1 antibody was used, this shows co-precipitation with AP-1A only at best and not pan-AP-1 as stated in the text.

Response: We had performed the experiments with appropriate controls. Here we have provided the controls in Fig.S4B. We have modified the text accordingly (line no. 257-259).

*****

<u>**Reviewer 3 Advance Summary and Potential Significance to Field:**</u>

This is a revised submission of a manuscript that describes polarized sorting of the copper ATPases ATP7A and ATP7B. The authors find that the AP1 complex is involved in the polarized distribution of these molecules with different roles for copper in directing the trafficking. They further identify motifs in the amino terminal domain of ATP7B that are mutated in human genetic disease.

Reviewer 3 Comments for the Author:
The authors have somewhat responded to the previous critique. I do have some questions about how the results are interpreted.

1. It is stated the ATP7A does not traverse an endosomal compartment. However, the colocalization of ATP7A with internalized transferrin is nearly the same as that seen with ATP7B and EEA1, which is stated as evidence for ATP7B traversing the ASE.

Response: The colocalization of internalized Tf with ATP7A is same as ATP7B with EEA1 at 15 mins time point of copper treatment, indicating very low enrichment (>0.2 in Mander's analysis, Fig. 3A, *bottom panel* and Fig. 3B) of ATP7B in EEA1 positive compartments. But as the treatment time increases (30, 45 and 60 mins) there is an increase in colocalization of ATP7B with EEA1 which indicates that ATP7B traverses through EEA1 compartments in response to copper. But that is not the case for ATP7A which stays the same and has low level of colocalization suggesting low enrichment in those compartments.

2. Since AP1B knockout results in normal targeting of both transporters, how can it be that, as stated in the abstract "AP-1B governs polarized trafficking of ATP7B solely."

Response: We appreciate the concern of the reviewer. We have modified the statement which fits our observations better (line no.36 in abstract).

3. While an endosomal trafficking route is discussed in the discussion, the manuscript focuses on

© 2023. Published by The Company of Biologists under the terms of the Creative Commons Attribution License (https://creativecommons.org/licenses/by/4.0/).

the role of AP1 in retention of these transporters in the TGN. I do not think that the data can distinguish between a role for AP1 in TGN retention and a failure to retrieve the transporters from the plasma membrane or endosomal compartments.

Response: We agree with the reviewer that it is very difficult to distinguish between retention and retrieval. We have modified the model which best aligns with our observations. We have modified the proposed model (Fig. 6G) by adding dotted double-headed arrows indicating TGN retention and also mentioned it in the discussion section (line 468-473).

4. *It is my opinion that the videos are superfluous.*

Response: According to the editor's remarks we have kept the videos in the manuscript.

Third decision letter

MS ID#: JOCES/2023/261258

MS TITLE: Regulation of apico-basolateral trafficking polarity of homologous Copper-ATPases ATP7A and ATP7B

AUTHORS: Arnab Gupta, Ruturaj ., Monalisa Mishra, Soumyendu Saha, Saptarshi Maji, Enrique Rodriguez-Boulan, and Ryan Schreiner
ARTICLE TYPE: Research Article

I am happy to tell you that your manuscript has been accepted for publication in Journal of Cell Science, pending standard ethics checks.

© 2023. Published by The Company of Biologists under the terms of the Creative Commons Attribution License (https://creativecommons.org/licenses/by/4.0/).
